# Photosynthetic Bacteria‐Hitchhiking 2D *i*MXene‐mRNA Vaccine to Enable Photo‐Immunogene Cancer Therapy

**DOI:** 10.1002/advs.202307225

**Published:** 2024-05-14

**Authors:** Shen Zhang, Jifeng Yu, Yunyun Liu, Bing Xiong, Yan Fang, Yuli Zhu, Shaoyue Li, Liping Sun, Boyang Zhou, Yikang Sun, Lifan Wang, Wenwen Yue, Haohao Yin, Huixiong Xu

**Affiliations:** ^1^ Department of Ultrasound Institute of Ultrasound in Medicine and Engineering Zhongshan Hospital Fudan University Shanghai 200032 P. R. China; ^2^ Department of Medical Ultrasound Center of Minimally Invasive Treatment for Tumor Shanghai Tenth People's Hospital Ultrasound Research and Education Institute Clinical Research Center for Interventional Medicine School of Medicine Tongji University. Shanghai 200072 P. R. China; ^3^ Department of Medical Ultrasound Shanghai Tenth People's Hospital Nanjing Medical University Shanghai 200072 P. R. China; ^4^ Shanghai Engineering Research Center of Ultrasound Diagnosis and Treatment Shanghai 200072 P. R. China

**Keywords:** gene cancer therapy, immunotherapy, mRNA vaccine, photosynthetic bacteria

## Abstract

Therapeutic mRNA vaccines have become powerful therapeutic tools for severe diseases, including infectious diseases and malignant neoplasms. mRNA vaccines encoding tumor‐associated antigens provide unprecedented hope for many immunotherapies that have hit the bottleneck. However, the application of mRNA vaccines is limited because of biological instability, innate immunogenicity, and ineffective delivery in vivo. This study aims to construct a novel mRNA vaccine delivery nanosystem to successfully co‐deliver a tumor‐associated antigen (TAA) encoded by the Wilms' tumor 1 (*WT1*) mRNA. In this system, named PSB@Nb_1.33_C/mRNA, photosynthetic bacteria (PSB) efficiently delivers the *i*MXene‐*WT1* mRNA to the core tumor region using photo‐driven and hypoxia‐driven properties. The excellent photothermal therapeutic (PTT) properties of PSB and 2D *i*Mxene (Nb_1.33_C) trigger tumor immunogenic cell death, which boosts the release of the *WT1* mRNA. The released *WT1* mRNA is translated, presenting the TAA and amplifying immune effect in vivo. The designed therapeutic strategy demonstrates an excellent ability to inhibit distant tumors and counteract postsurgical lung metastasis. Thus, this study provides an innovative and effective paradigm for tumor immunotherapy, i.e., photo‐immunogene cancer therapy, and establishes an efficient delivery platform for mRNA vaccines, thereby opening a new path for the wide application of mRNA vaccines.

## Graphical abstract

The light‐driven and hypoxia‐driven properties are used to deliver Wilms' tumor 1 (*WT1*) mRNA to the tumor region. The photothermal therapeutic property of bacteria and 2D *i*Mxene triggers immune cell death and release of *WT1* mRNA. The primary and distant tumors are inhibited and lung metastasis is prevented, providing an innovative paradigm for photo‐immunogene cancer therapy.

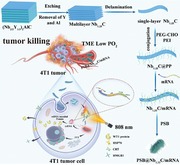

## 1 Introduction

Recently, mRNA vaccines have attracted significant attention as promising therapeutic tools for treating tumors.^[^ ^1^ ^]^ mRNA vaccines can encode several tumor‐associated antigens (TAAs), and intracellular protein translation and antigen processing within the cells result in the formation of complexes with major histocompatibility complex I (MHC I) in antigen‐presenting cells (APCs), primarily dendritic cells (DCs), which subsequently present antigens to T cells, thereby triggering a robust tumor‐specific T cell response.^[^ ^2^ ^]^ The Wilms’ tumor gene, *WT1*, is involved in the pathogenesis of different types of cancers.^[^ ^3^ ^]^ The WT1 protein naturally participates in regulating the humoral and cellular immunity in patients with cancer, which is indicative of its immunogenicity. Thus, cancer immunotherapies targeting *WT1* would be valuable.^[^ ^4^ ^]^ However, the application of mRNA vaccines has been restricted because of their biological instability, large molecular weight, high intrinsic immunogenicity, and insufficient delivery in vivo.^[^ ^5^ ^]^ Therefore, these limitations should be overcome to improve the use of mRNA vaccines.

2D materials resemble plate‐like nanomaterials with a high lateral size‐to‐thickness ratio, excellent flexibility, and tunable physicochemical properties.^[^ ^6^ ^]^ The 2D nanomaterials hold great potential in cancer applications.^[^ ^7^ ^]^ 2D nanomaterials possess considerable potential for application in the field of cancer therapy. The extra‐large surface areas of 2D materials can efficiently load several functional molecules, including fluorescent probes, chemotherapeutic drugs, and biomacromolecules, via covalent or non‐covalent mutual interactions.^[^ ^8^ ^]^ The surface of the layered structure of MXene, a 2D nanomaterial, can be utilized to load many therapeutic agents, including chemotherapeutic drugs and biological macromolecules.^[^ ^9^ ^]^ A new member of the MXene family with in‐plane ordered double‐transition metals, named *i*MXene,^[^ ^10^ ^]^ has been investigated recently.^[^ ^11^ ^]^ The *i*MXene system, (M″_2/3_ M′_1/3_) X, includes the novel in‐plane ordered double‐transition metals in the MAX phase, and the chemical formula is (M″ M′) AX.^[^ ^12^ ^]^ *i*MXene has excellent transport capability, as demonstrated by studies on its electronic properties.^[^ ^11b^ ^]^ *i*MXene is mainly produced by selective etching of elements and further delamination to produce a single‐layer nanosheet.^[^ ^11^, ^13^ ^]^ Based on studies on *i*MXene, Nb_1.33_C nanosheets were constructed and applied in cancer therapy as both mRNA vaccine delivery vehicles and photothermal biomaterials to kill tumor cells and induce long‐term immunity. However, nanomaterials do not successfully accumulate at diseased sites, and biological barriers limit effective responses in diseases such as cancer and inflammation.^[^ ^14^ ^]^ Therefore, precise targeted delivery of mRNA vaccines to tumor cells is challenging.

In recent decades, certain live microorganisms have been demonstrated to efficiently deliver drug‐loaded substances into hypoxic areas of tumor tissues because of their ability to migrate in the presence of light and magnetism or their self‐driving nature. Moreover, certain microorganisms can directly attack cancer cells and act as therapeutic agents.^[^ ^15^ ^]^ Live microorganisms have been used to counter tumors in sonodynamic and photodynamic therapies,^[^ ^16^ ^]^ and facultative anaerobes such as photosynthetic bacteria (PSB) possess the properties of an original photoenergy synthesis system.^[^ ^17^ ^]^ The ability of a living PSB to adsorb light in the near‐infrared (NIR) region allows it to act as a photothermal agent, generating heat. The synergistic combination of photothermal and gene therapy demonstrates great potential for breast cancer.^[^ ^18^ ^]^ The synergistic combination of photothermal therapy (PTT) and gene therapy may be potentially used for treating breast cancer. Using the combination of its hypoxia‐targeting and PTT properties, PSB can achieve hypoxia‐targeted cancer therapy without any modification.^[^ ^19^ ^]^ Therefore, PSB may be used as a delivery tool for targeting tumor sites and for killing tumor cells as photothermal therapeutic agents under NIR laser irradiation to enhance the activation of the immune system.

**Figure 1:**
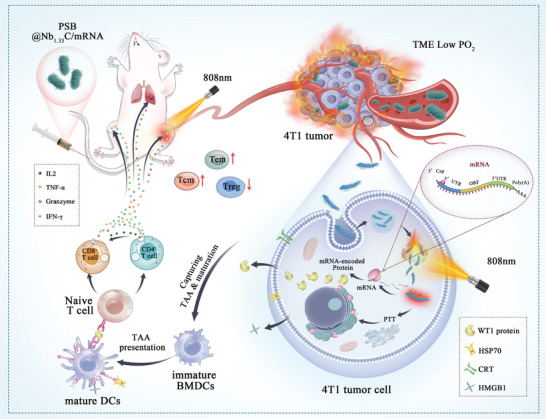
Schematic showing the design of the 808 nm NIR laser‐mediated delivery mRNA vaccine for activating the immune system in tumors. The process of 808 nm NIR laser‐mediated delivery of the mRNA vaccine to the nuclei of tumor cells. Ribosomes can read the mRNA after internalization and transition to the cytosol and then translate it into proteins that undergo post‐translational modifications, forming a properly folded functional protein that upregulates the expression of tumor‐associated antigens. In addition, the photothermal therapeutic effect can kill tumor cells via immunogenic cell death. The PSB@Nb_1.33_C/mRNA delivery system achieved tumor killing, activation of the immune system, and establishment of long‐term immune memory for cancer therapy.

In this study, we aimed to design a novel mRNA vaccine delivery system based on PSB@Nb_1.33_C/mRNA and investigated its ability to inhibit 4T1 primary and distant tumor progression and prevent lung metastasis by activating the immune system in vivo (**Figure** **1**). Due to its outstanding hypoxia‐targeting property, living PSB were used to efficiently and safely deliver the system. The 808 nm NIR laser was applied at the tumor site after enriching the tumor with the PSB@Nb_1.33_C/mRNA delivery system. PSB and Nb_1.33_C exhibited photothermal properties and triggered immunogenic cell death (ICD) to activate the immune system and achieve tumor‐killing. Furthermore, translation of the *WT1* mRNA occurred in tumor cells, which considerably improved the expression of the WT1 protein. A powerful immune system response was induced by the upregulation of WT1 and TAAs mediated by the ICD of tumor cells, which caused further DCs maturation. Cytotoxic T lymphocytes (CTL) and inflammatory cytokines were upregulated to inhibit tumor growth, metastasis, and recurrence. The designed delivery vehicle exhibited powerful immune activation, damaging 4T1 tumor tissue and inhibiting tumor metastasis in mice in vivo. Furthermore, the effective immune memory effect produced by the nanosystem strongly prevented the lung metastasis of 4T1 tumor cells. We believe this study will provide a novel delivery nanocarrier for mRNA vaccines and an anti‐tumor therapeutic strategy that can be widely applied in cancer therapy.

## 2 Results

### 2.1 Design and Characterizations of PSB@Nb_1.33_C for the Delivery of *WT1* mRNA Cancer Vaccine

**Figure 2:**
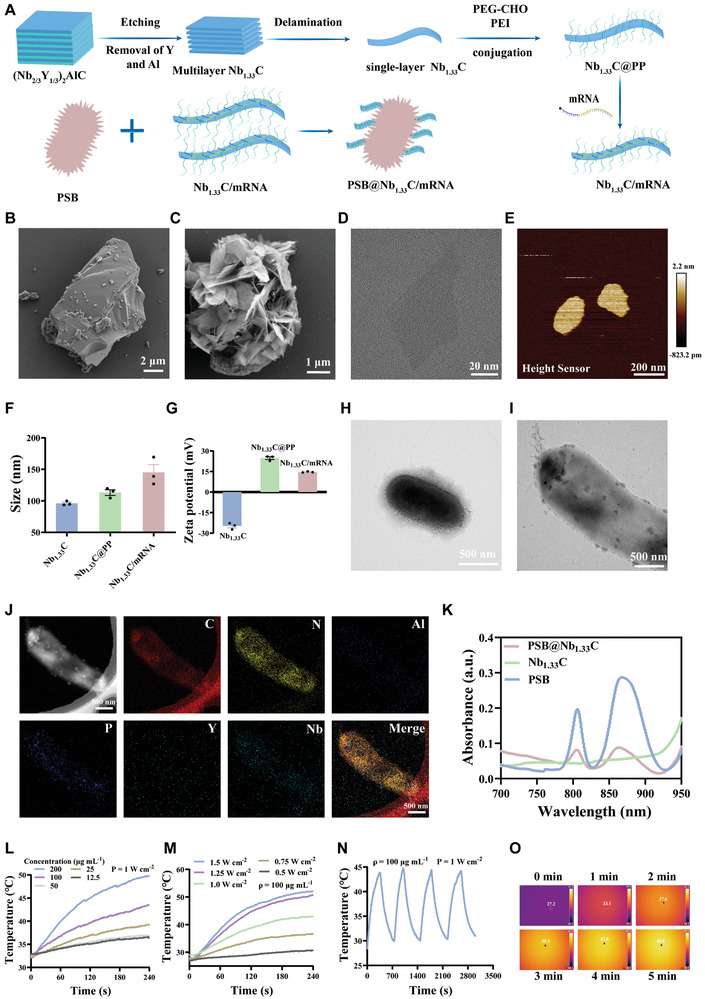
Synthesis, structure, and photothermal characteristics of PSB@Nb_1.33_C/mRNA. A) Synthesis of PSB@Nb_1.33_C/mRNA. B) SEM of (Nb_2/3_Y_1/3_)_2_AlC and C) multilayer Nb_1.33_C. D) TEM of Nb_1.33_C single‐layer nanosheet. E) Typical AFM image of the Nb_1.33_C nanosheet. F) Particle size of Nb_1.33_C, Nb_1.33_C@PP, and Nb_1.33_C/mRNA (*n* = 3). G) Zeta potential of Nb_1.33_C, Nb_1.33_C@PP, and Nb_1.33_C/mRNA (*n* = 3). H) TEM of living PSB. I) TEM and J) the corresponding element mapping images (C, N, Al, P, Y, and Nb elements) of PSB@Nb_1.33_C/mRNA. K) UV‐vis absorption spectra of Nb_1.33_C, PSB, and PSB@Nb_1.33_C. L) Photothermal temperature curves of PSB@Nb_1.33_C (PSB = 10^9^ CFU mL^−1^) at different concentrations of Nb_1.33_C (1 W cm^−2^ for 808 nm laser irradiation). M) The heating curves of PSB@Nb_1.33_C (PSB = 10^9^ CFU mL^−1^, Nb_1.33_C = 100 µg mL^−1^) under various power densities. N) Temperature change in PSB@Nb_1.33_C (PSB = 10^9^ CFU mL^−1^, Nb_1.33_C = 100 µg mL^−1^) with four irradiation and cooling periods (1 W cm^−2^ for 808 nm laser irradiation). O) Corresponding thermal images of PSB@Nb_1.33_C (PSB = 10^9^ CFU mL^−1^, Nb_1.33_C = 100 µg mL^−1^) after irradiation with 1 W cm^−2^ 808 nm laser.

To construct a nanosystem for *WT1* mRNA cancer vaccine delivery, monolayer nanosheets of Nb_1.33_C were prepared using liquid‐phase exfoliation. The mPEG‐d‐PEI‐coated Nb_1.33_C was prepared by adding Nb_1.33_C to the mPEG‐d‐PEI solution and stirring for 30 min at room temperature. The *WT1* mRNA was prepared using an in vitro transcriptional strategy. mPEG‐PEI‐coated Nb_1.33_C (Nb_1.33_C@PP) and the prepared *WT1* mRNA were mixed. Next, the Nb_1.33_C‐loaded mRNA solution was added to the activated living PSB suspension at room temperature and stirred for 30 min. Finally, a PSB@Nb_1.33_C/mRNA nanosystem was obtained (**Figure** **2**A). Representative scanning electron microscopy (SEM) images showed the characterization of (Nb_2/3_Y_1/3_)_2_AlC and multilayer Nb_1.33_C (Figure 2B,C). These results suggested the production of multilayer Nb_1.33_C after HF etching. The corresponding element mapping images of the nanomaterial have been provided (Figure S1A,B, Supporting Information). This indicated that Y and Al were selectively removed to a considerable extent after HF‐mediated etching. The Nb_1.33_C single nanosheets were investigated using transmission electron microscopy (TEM). After delamination of the TBAOH solution, Nb_1.33_C single‐layer nanosheets with vertical size of ≈100 nm were prepared (Figure 2D). Furthermore, the corresponding element mapping images of the Nb_1.33_C nanosheets were obtained to analyze the changes in the elements during the preparation of the Nb_1.33_C single‐layer nanosheet (Figure S1C, Supporting Information). The results demonstrated that Nb and C were retained, whereas Y and Al were removed significantly. Atomic force microscopy (AFM) was used to determine the thickness of the Nb_1.33_C nanosheets (Figure 2E). The apparent average thickness of the Nb_1.33_C nanosheets was 1.113 nm. In addition, the particle sizes of Nb_1.33_C, Nb_1.33_C@PP, and Nb_1.33_C/mRNA were investigated using dynamic light scattering (DLS), which revealed that the average diameters of Nb_1.33_C and Nb_1.33_C@PP were 96.11 and 113.20 nm, respectively. The average diameter of Nb_1.33_C increased to 145.13 nm when the mRNA loading was successful (Figure 2F). The polydispersity index (PDI) and DLS analysis of Nb_1.33_C/mRNA within 14 days were used to investigate its dispersion stability (Figure S2, Supporting Information). The size of Nb_1.33_C/mRNA showed a slight increase of only 7.8% and 9.4% after 7 and 14 days, respectively, indicating good stability. Furthermore, the PDI values (0.18‐0.23) of Nb_1.33_C/mRNA remained unchanged after 7 and 14 days, indicating further stability of the system. In addition, zeta potential analysis was used to further confirm the successful loading of the mRNA on Nb_1.33_C (Figure 2G). The results indicated that the average Nb_1.33_C zeta potential was −24.74 mV and that modification with mPEG‐d‐PEI reversed the Nb_1.33_C zeta potential by 24.79 mV. Nb_1.33_C/mRNA showed a relatively reduced potential because of the negative charge of the mRNA, which further indicated successful loading of the mRNA. The TEM images of PSB and PSB@Nb_1.33_C/mRNA were used for characterization (Figure 2H,I). Compared with PSB, PSB@Nb_1.33_C/mRNA showed certain sheet‐like attachments on the PSB surface in TEM, indicating that living PSB can load nanosheets during the preparation methods. In addition, the corresponding element mapping images (C, N, Al, P, Y, and Nb) of PSB and PSB@Nb_1.33_C/mRNA were obtained to investigate the changes in element composition (Figure 2J; Figure S1D, Supporting Information). The Nb content in PSB@Nb_1.33_C/mRNA was distributed unevenly, unlike that in PSB. The NanoDrop was used to determine the mRNA loading on the bacterial surface. Prior to preparing the Nb_1.33_C/mRNA, we tested RNA concentration of PSB, and the concentration of mRNA solution was explored as the control group. After preparing the PSB@Nb_1.33_C/mRNA, the mRNA concentration was measured at different time points to determine the stability of Nb_1.33_C/mRNA coupled to the bacterial surface. The results showed that there is a large mount mRNA loading on the PSB bacterial surface. Besides, the RNA concentration had only slightly decreased within 24 h, suggesting the excellent stability of the Nb_1.33_C/mRNA on the PSB surface (Figure S3, Supporting Information). In addition, the UV‐vis absorption spectra of various concentrations of Nb_1.33_C and the absorption peaks of PSB were analyzed (Figure S4A, B, Supporting Information). The differences in the UV‐vis absorption spectra of Nb_1.33_C, PSB, and PSB@Nb_1.33_C suggested that the absorption peaks of PSB@Nb_1.33_C were following those of living PSB (Figure 2K). This demonstrated that the Nb_1.33_C nanosheets were successfully loaded on the PSB delivery carrier. The thermogravimetric analysis of Nb_1.33_C and mPEG‐Nb_1.33_C‐PEI were applied to investigate the loading capacity of mPEG‐d‐PEI. The thermogravimetric analysis determined that the mPEG‐d‐PEI in mPEG‐Nb_1.33_C‐PEI was ≈ 23.98%(Figure S5, Supporting Information). To investigate the ideal loading efficiency of Nb_1.33_C/mRNA, Nb_1.33_C nanosheets and *WT1* mRNA (Nb_1.33_C/mRNA) were mixed in different mass ratios, and the supernatants were collected after co‐incubation and centrifugation. Agarose gel electrophoresis was used to detect the loading efficiency (Figure S6, Supporting Information). The results indicated that the supernatant contained mRNA very slightly at the loading efficiency of 4:1, suggesting a relatively satisfactory loading effect for mRNAs at that ratio. Thus, the PSB@Nb_1.33_C/mRNA nanosystem was prepared successfully.

### 2.2 Photothermal effect of the PSB@Nb_1.33_C nanosystem

The photothermal performances of PSB and Nb_1.33_C were individually investigated to demonstrate their heating effects (Figure S7A–F, Supporting Information). The results indicated that living PSB and Nb_1.33_C nanosheets can inherently convert light to heat. PSB@Nb_1.33_C showed an absorption peak in the 800−850 nm range. The PSB and Nb_1.33_C nanosheets exhibited photothermal conversion when irradiated with the 808 nm NIR laser. The photothermal properties of the delivery nanosystem were determined after irradiation with the 808 nm laser. The temperature changes of PSB@Nb_1.33_C are shown in Figure 2L, which suggests that the photothermal performance increased with PSB@Nb_1.33_C concentration. The temperature of PSB@Nb_1.33_C increased to 43.6 °C after 4 min of irradiation with the 808 nm NIR laser. In addition, the heating curve was investigated at different power densities (Figure 2M); the results revealed that the nanosystem exhibited a heating effect at various laser radiation power densities, which was indicative of the controllable photothermal effect of the PSB@Nb_1.33_C delivery system. Furthermore, temperature changes were detected and recorded during the four irradiation and cooling periods to investigate the photothermal stability (Figure 2N). The photothermal performance did not change significantly during these periods. The corresponding thermal images of PSB@Nb_1.33_C are shown in Figure 2O. These results indicated that the PSB@Nb_1.33_C delivery system may be used for killing tumor cells via the photothermal effect.

### 2.3 Effectiveness of the mRNA nanovaccine In Vitro

**Figure 3:**
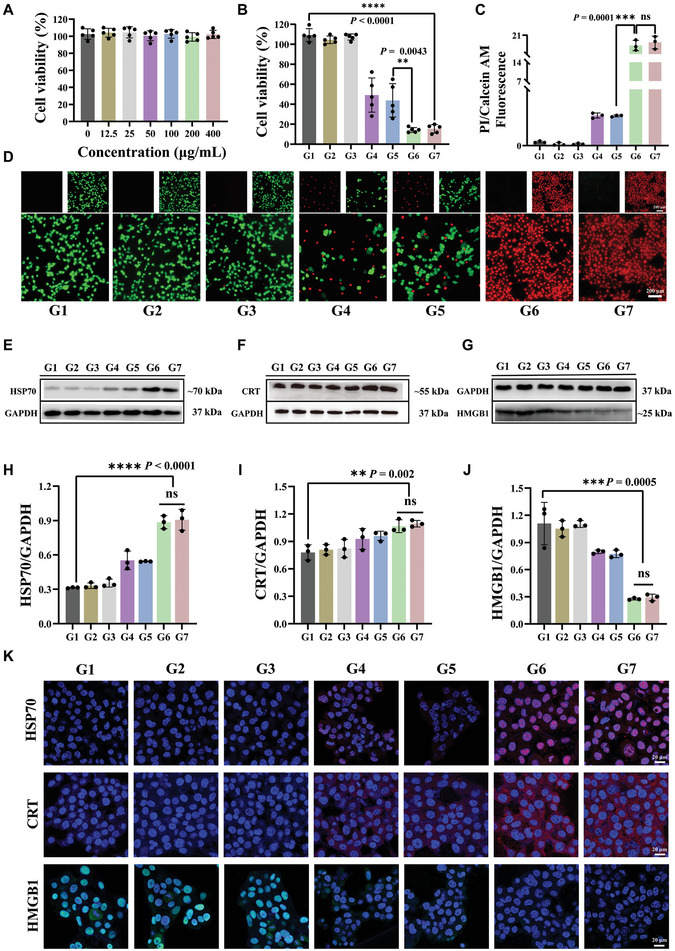
In vitro therapeutic efficacy of PSB@Nb_1.33_C/mRNA against 4T1 tumor cells. A) Viability of 4T1 tumor cells co‐incubated with various concentrations of Nb_1.33_C/mRNA for 12 h (Nb_1.33_C = 0, 12.5, 25, 50, 100, 200 and 400 µg mL^−1^) (*n* = 5). B) Viability of 4T1 tumor cells in different groups (*n* = 5). C) Corresponding fluorescence intensity of propidium iodide (PI)/calcein‐AM of 4T1 tumor cells after various treatments (*n* = 3). D) PI/calcein‐AM fluorescence image for different groups. E‐G) Western blot analysis of DAMP‐related protein expression (HSP70, CRT, HMGB1) in various groups. H‐J) The corresponding quantitative analysis of HSP70, CRT, and HMGB1 using western blotting (*n* = 3). *p <0.05, **p <0.01, ***p <0.001, ****p <0.0001. K) Immunofluorescence images of HSP70, CRT, and HMGB1 in 4T1 tumor cells of various groups. Control (G1), PSB@Nb_1.33_C/mRNA (G2), laser (G3), Nb_1.33_C/mRNA + laser (G4), PSB + laser (G5), PSB@Nb_1.33_C + laser (G6), PSB@Nb_1.33_C/mRNA + laser (G7).

After verifying its ability to load mRNA and the PTT properties, we investigated the therapeutic efficacy of PSB@Nb_1.33_C/mRNA. Firstly, the optimal cellular uptake time point was determined using confocal laser scanning microscopy (CLSM) after co‐incubation of Nb_1.33_C/mRNA with 4T1 tumor cells for different durations (Figure S8A,B, Supporting Information). The results showed that Cy3‐labeled Nb_1.33_C/mRNA mostly entered tumor cells after co‐incubation for 8 h. The mean fluorescence intensity analysis showed no significant difference between 8 and 10 or 12 h, indicating that the peak was reached at 8 h. We used a confocal microscope to capture high‐magnification images and explore whether mRNA could escape the lysosome (Figure S9A–D, Supporting Information). The results indicated that the Nb_1.33_C/mRNA could be largely detected in 4T1 cells with strong red fluorescence signals under the 808 nm NIR laser, the corresponding fluorescence intensity and confocal imaging study also indicated that mRNA could be efficiently delivered into the cytoplasm of cells. GFP‐mRNA was used to explore the uptake and translation of mRNA by different cells in tumor tissue. The GFP‐mRNA was taken up by 4T1 tumor cells, cancer‐associated fibroblasts (CAF), and tumor‐associated macrophages (TAM) in the single‐cell phenotype model in vitro (Figure S10A,B, Supporting Information). The results showed that under the 808 nm NIR laser, the GFP mRNA vaccine can translate in the 4T1, CAF, and TAM(RAW 264.7) cells. In addition, flow cytometry (FCM) was used to explore the proportion of GFP^+^ tumor cells in vitro (Figure S10C,D, Supporting Information). The results indicated that the proportion of GFP cells apparently upregulated in the group of PSB@Nb_1.33_C/mRNA + laser, which suggests the efficacy of the designed mRNA nanovaccine delivery system. 4T1 cells were co‐incubated with varying concentrations of Nb_1.33_C/mRNA. As shown in **Figure** **3**A, the results obtained using the standard cell counting kit‐8 (CCK‐8) assay demonstrated that Nb_1.33_C/mRNA had satisfactory biosafety. The 4T1 tumor cells were then treated with different regimens, which included the Control, PSB@Nb_1.33_C/mRNA, laser, Nb_1.33_C/mRNA + laser, PSB + laser, PSB@Nb_1.33_C + laser, PSB@Nb_1.33_C/mRNA + laser. The results of the CCK‐8 assay indicated the ability of Nb_1.33_C/mRNA and PSB to kill tumor cells after 808 nm NIR laser irradiation. The tumor cell‐killing effect of the PSB@Nb_1.33_C/mRNA + laser group, with average cell viability of 15.43%, was more significant than that of the Nb_1.33_C/mRNA + laser or PSB + laser groups, with average cell viabilities of 49.16% and 43.89%, respectively, indicating that the nanosystem exerted a favorable PTT effect by attacking the tumor cells (Figure 3B). Subsequently, the condition of live/dead cells was investigated using calcein acetoxymethyl ester (calcein‐AM) and propidium iodide (PI) staining (Figure 3C,D). An evident red fluorescence intensity was observed in the PSB@Nb_1.33_C + laser and PSB@Nb_1.33_C/mRNA + laser groups compared to those in the other treatment groups, revealing the ability of the designed nanosystem to kill the tumor cells. FCM was used to investigate apoptosis; the results revealed that the PSB@Nb_1.33_C/mRNA+ laser group showed considerable apoptosis (Figure S11, Supporting Information), suggesting a significant increase in the apoptosis ratio compared to that in the control group.

Western blot and immunofluorescence staining were used to investigate the ICD of 4T1 cells induced by the PSB@Nb_1.33_C/mRNA + laser treatment. Calreticulin (CRT), high‐mobility group box 1 (HMGB1), and heat shock protein 70 (HSP70) are considered typical ICD biomarkers. As observed in western blot, the 4T1 cells treated with PSB@Nb_1.33_C + laser and PSB@Nb_1.33_C/mRNA + laser showed upregulation of CRT and HSP70 and downregulation of HMGB1 compared to those in the other groups, suggesting that ICD effected in PSB@Nb_1.33_C and PSB@Nb_1.33_C/mRNA in the presence of the NIR laser (Figure 3E–J). The results were verified using CLSM. The CLSM images indicated that the fluorescence intensities of CRT and HSP70 in PSB@Nb_1.33_C + laser and PSB@Nb_1.33_C/mRNA + laser groups were stronger than those in the other groups. The fluorescence intensity of HMGB1 showed the opposite trend (Figure 3K; Figure S12, Supporting Information). These results indicated that the nanosystem can achieve effective tumor‐killing effects via ICD. This has the potential to promote DCs maturation and activate the immune system.

### 2.4 Expression of *WT1* mRNA and DC Maturation in vitro

**Figure 4:**
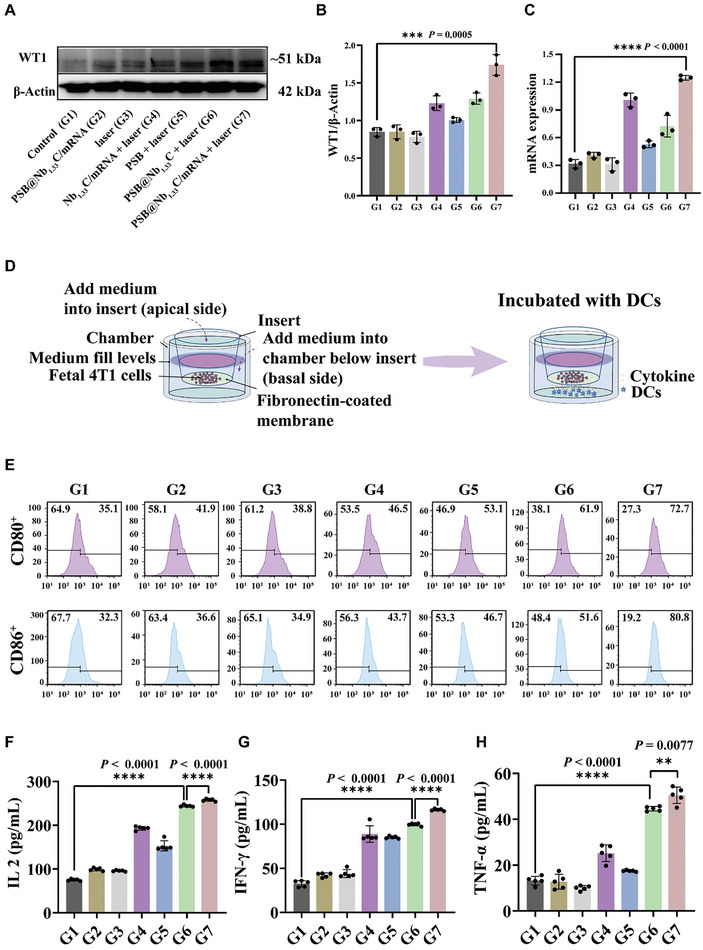
Expression of *WT1* mRNA and protein, activation of the immune response, and maturation of DCs after treatment with the mRNA nanovaccine in vitro. A) Western blot analysis of WT1. B) Quantitation of WT1 in western blotting. C) qRT‐PCR analysis of *WT1* mRNA in the 4T1 tumor cells of different groups. D) Schematic illustration of the experiment used to assess DC maturation using the transwell system. 4T1 tumor cells were treated using various regimens in the upper chamber, while the BMDCs were cultured in the lower chamber. After 24 h, the BMDCs were assembled and analyzed. The cytokines detected in the supernatant included IL 2, IFN‐γ, and TNF‐α. E) After different treatments, the typical FCM and corresponding statistical data of DCs (CD80^+^ CD86^+^ CD11c^+^) (*n* = 3). F‐H) IL 2, IFN‐γ, and TNF‐α expression levels in the supernatant of BMDCs. (*n* = 5) Control (G1), PSB@Nb_1.33_C/mRNA (G2), laser (G3), Nb_1.33_C/mRNA + laser (G4), PSB + laser (G5), PSB@Nb_1.33_C + laser (G6), PSB@Nb_1.33_C/mRNA + laser (G7).

The expression levels of the WT1 protein and *WT1* mRNA were verified using western blotting and quantitative real‐time polymerase chain reaction (qRT‐PCR), respectively. WT1 protein and *WT1* mRNA expression levels were significantly elevated after treatment with PSB@Nb_1.33_C/mRNA + laser (**Figure** **4**A–C). These results indicated that the nanovaccine carried by PSB@Nb_1.33_C increased TAA expression and could activate the anti‐tumor immune system.

After confirming that this delivery system enabled high expression of the *WT1* mRNA and protein in vitro, the ability of this therapeutic strategy to activate the immune system was investigated. As the most promising APCs, DCs can activate and regulate naive and memory immune responses.^[^ ^20^ ^]^ The transwell system was used to investigate the maturation of DCs and the related characteristic cytokine expression (Figure 4D). DCs were cultured with 4T1 tumor cells after various treatments. The results of FCM indicated that the 4T1 tumor cells processed by the PSB@Nb_1.33_C/mRNA nanosystem under NIR laser irradiation significantly promoted DC maturation compared to that observed in the PSB@Nb_1.33_C + laser group (Figure 4E; Figure S13, Supporting Information). Furthermore, the supernatants from the cell culture were collected. The enzyme‐linked immunosorbent assay (ELISA) was performed. The results indicated that a series of pro‐inflammatory cytokines such as interleukin‐2 (IL 2), interferon‐γ (IFN‐γ), and tumor necrosis factor‐α (TNF‐α) were upregulated in the PSB@Nb_1.33_C/mRNA + laser group (Figure 4F–H). All the results demonstrated that the PSB@Nb_1.33_C/mRNA delivery system possesses considerable potential for inducing the innate immune system after irradiation with the 808 nm NIR laser and that it further effectively induced DC maturation to activate the adaptive immune system and attack tumor cells.

### 2.5 Effectiveness of the Nanosystem in Treating Primary and Distant Tumors In Vivo

Firstly, the biosafety of PSB@Nb_1.33_C/mRNA was evaluated using hematoxylin and eosin (HE) staining and blood biochemical analysis following the administration of PSB@Nb_1.33_C/mRNA via the tail veins of mice. A comprehensive analysis of the blood biochemical markers in the treated mice, including red blood cell, white blood cell, platelet counts, and liver and kidney functions, indicated that the results did not differ significantly between the various time points (Figure S14, Supporting Information). HE staining was performed on major organs, including the heart, liver, spleen, lungs, and kidneys, at various time intervals. Compared to the organs of the control group, obvious inflammation or necrosis was not detected (Figure S15, Supporting Information). These results suggested that the delivery nanosystem was highly biosafe and biocompatible.

**Figure 5:**
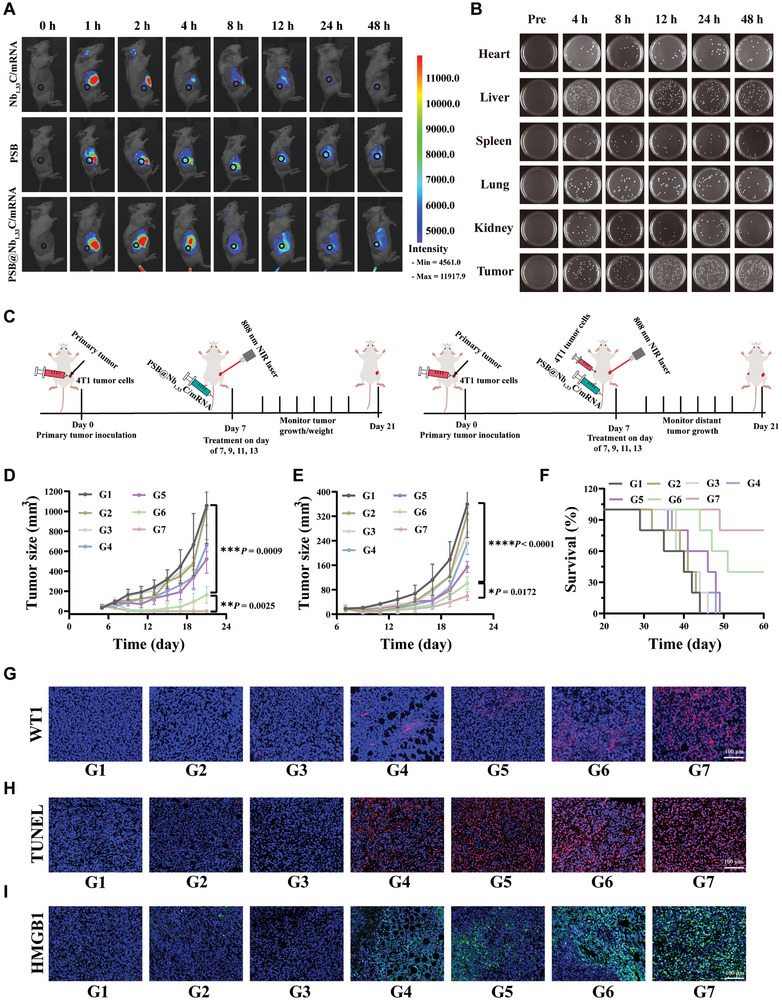
Activity of PSB@Nb_1.33_C/mRNA against primary and distant tumors in vivo. A) In vivo imaging of IR783‐labeled Nb_1.33_C/mRNA, PSB, and PSB@Nb_1.33_C/mRNA in mice. B) Typical images of solid ATYP agar plates showing bacterial colonization of 4T1 tumor‐bearing mice at different time points after injection of PSB (10^8^ CFU per mouse, *n* = 3). C) Schematic showing establishment of in vivo primary and distant tumor models. D) Average 4T1 primary tumor growth curves (*n* = 5). E) Average 4T1 distant tumor growth curves (*n* = 5). *p <0.05, **p <0.01, ***p <0.001, ****p <0.0001. F) Survival rates of the 4T1 tumor‐bearing mice in different groups. G) Corresponding images of WT1 immunofluorescence staining for the 4T1 primary tumor tissue. H) TUNEL immunofluorescence staining images of 4T1 primary tumor tissue. I) Immunofluorescence images of HMGB1 in primary tumor tissue after different treatments. Control (G1), PSB@Nb_1.33_C/mRNA (G2), laser (G3), Nb_1.33_C/mRNA + laser (G4), PSB + laser (G5), PSB@Nb_1.33_C + laser (G6), PSB@Nb_1.33_C/mRNA + laser (G7).

Next, the hypoxia‐targeting ability of the nanosystem was investigated. A biodistribution assay of PSB@Nb_1.33_C/mRNA was performed in the experimental model of BALB/c mice bearing 4T1 tumors. Nb_1.33_C/mRNA, PSB, and PSB@Nb_1.33_C/mRNA were administered to mice via intravenous injection (PSB = 10^9^ colony‐forming unit (CFU) mL^−1^, Nb_1.33_C = 100 µg mL^−1^) when the tumor volume reached ≈200 mm^3^. Nb_1.33_C/mRNA, PSB, and PSB@Nb_1.33_C/mRNA labeled with the NIR fluorescent dye, IR783, were used to investigate their tumor‐targeting ability. Fluorescence imaging was performed, and the corresponding fluorescence intensities were observed at different time points (**Figure** **5**A; Figure S16, Supporting Information). The results suggested that the fluorescence intensity of IR783 increased gradually, indicating the tumor‐targeting ability of the mRNA delivery system. In addition, IR783 labeled mRNA was used to analyze the distribution in tumors and major organs (heart, liver, spleen, lung, and kidney) over time. The tumors and major organs were harvested for fluorescence imaging at 48 h of injection of PBS, IR783 labeled Nb_1.33_C/mRNA and IR783 labeled PSB@Nb_1.33_C/mRNA(Figure S17A,B, Supporting Information). The results suggested that accumulated significantly stronger signals in tumors than the other organs in the PSB@Nb_1.33_C/mRNA groups, which demonstrated excellent tumor targeting properties of PSB@Nb_1.33_C/mRNA. Following PSB administration, the major organs and tumor tissues of mice were collected, homogenized, serially diluted, and spread on ATYP agar plates at different time points (Figure 5B). The CFUs on each agar plate were counted. The results indicated that PSB was gradually lost from the heart, liver, spleen, lungs, and kidneys. In contrast, the CFU increased in tumor tissues with time (Figure S18, Supporting Information). Thus, we concluded that the PSB@Nb_1.33_C/mRNA delivery system can target tumor sites and efficiently load Nb_1.33_C/mRNA.

Encouraged by the biosafety and hypoxia‐targeting ability of the PSB@Nb_1.33_C/mRNA nanosystem, a 4T1 tumor‐bearing BALB/c mouse model was established to investigate the tumor inhibition effect in vivo (Figure 5C). Mice were randomly allocated into seven groups, and the 4T1 tumor cells were injected and allowed to grow for 7 days. The mice received the following treatments: Control, PSB@Nb_1.33_C/mRNA, laser, Nb_1.33_C/mRNA + laser, PSB + laser, PSB@Nb_1.33_C + laser, and PSB@Nb_1.33_C/mRNA + laser. The 808 nm laser irradiated the tumor site for 5 min at a power density of 1 W cm^−2^. Tumor volumes were measured every 2 days (Figure 5D; Figure S19, Supporting Information). After 14 days of observation, tumor growth was observed to be significantly delayed in the PSB@Nb_1.33_C + laser and PSB@Nb_1.33_C/mRNA + laser groups compared to that in the other groups. However, the tumors in the PSB@Nb_1.33_C/mRNA + laser group disappeared completely. Simultaneously, a distant tumor mouse model was established to detect distant tumor inhibition. After the primary tumor was injected and sustained growth was observed for seven days, 1 × 10^6^ 4T1 tumor cells were injected on the opposite side of the primary tumor as a distant tumor. The mice were randomly allocated to seven groups, which received the same treatment as in the primary tumor experiment. Distant tumor volumes were monitored and recorded every 2 days for 21 days after the distant tumor cells were injected. The average distant tumor growth curve showed that tumor progression was significantly delayed in mice treated with PSB@Nb_1.33_C/mRNA + laser (Figure 5E; Figure S20 Supporting Information). This demonstrated that the treatment affected the distant tumors favorably in vivo. The survival curve indicated that the PSB@Nb_1.33_C/mRNA + laser treatment significantly prolonged mouse survival (Figure 5F). The body weights of these mice did not change significantly after the different treatments in each group, indicating the lack of systemic toxicity. (Figure S21, Supporting Information). Moreover, the WT1 protein level was detected using immunofluorescence in the tumor tissue. The results showed that the PSB@Nb_1.33_C/mRNA delivery system could achieve high levels of TAA expression (Figure 5G), indicating that the designed nanosystem realized the delivery of mRNA vaccines to increase the expression of TAAs that activated the anti‐tumor immune effects. Furthermore, the application of HE and TUNEL staining confirmed the pathological changes within the tumor tissue, indicating that massive cell necrosis occurred in the PSB@Nb_1.33_C/mRNA + laser group (Figure 5H; Figure S22A, Supporting Information). Moreover, cell proliferation was detected within the tumor using Ki‐67 staining, indicating that tumor cell proliferation was significantly inhibited in the PSB@Nb_1.33_C/mRNA + laser group compared to that in the other treated groups (Figure S22B, Supporting Information). In summary, these experiments demonstrated the efficient tumor‐killing effect of the nanosystem. In addition, the ICD effect induced by the different treatments was investigated using immunostaining of tumor sections for HMGB1, CRT, and HSP70 (Figure 5I; Figure S23A–C, Supporting Information), and the corresponding fluorescence intensities were determined (Figure S23D–F, Supporting Information). The results revealed that damage‐associated molecular pattern‐related proteins (DAMPs) were relatively highly expressed in the PSB@Nb_1.33_C/mRNA + laser group than in the other treatment groups. This implied that the proposed treatment strategy can effectively increase the ICD products. These results indicated that the designed mRNA vaccine delivery nanosystem exhibited targeted localization and enrichment at the tumor site, thereby exerting a powerful effect on tumor treatment.

### 2.6 Specific Immune Stimulation by PSB@Nb_1.33_C/mRNA Nanovaccines

**Figure 6:**
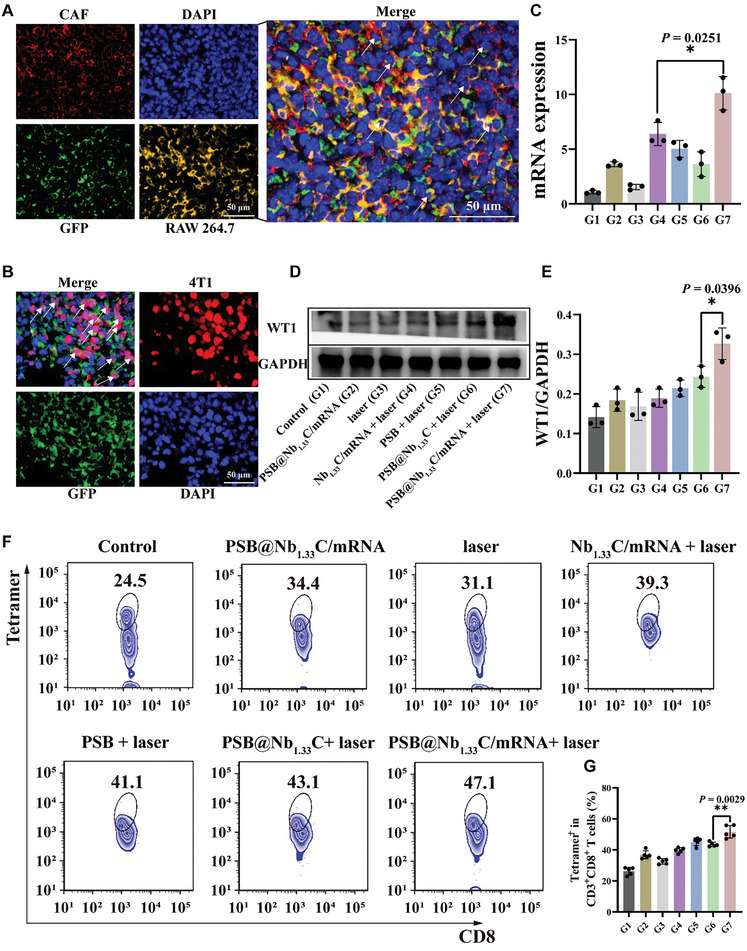
Specific immune stimulation by PSB@Nb_1.33_C/mRNA nanovaccines. A) Immunofluorescence images of GFP, CAF, and RAW 264.7. The CAF and RAW 264.7 were respectively characterized as α‐SMA positive and F4/80 positive in tumor tissue after the treatment of PSB@Nb_1.33_C/mRNA + laser. B) Immunofluorescence images of GFP, 4T1 (Ki‐67 positive) in tumor tissue after the treatment of PSB@Nb_1.33_C/mRNA + laser. C) qRT‐PCR analysis of *WT1* mRNA in the 4T1 tumor cells of different groups in vivo (*n* = 3). D) Western blot analysis of WT1 protein for the primary tumor tissue in vivo. E) Quantitation of WT1 protein in western blotting in vivo (*n* = 3). F) Typical FCM showing Tetramer for WT1‐specific CD8^+^ T cells and G) quantitative analysis of 4T1 tumor tissue (*n* = 5). Control (G1), PSB@Nb_1.33_C/mRNA (G2), laser (G3), Nb_1.33_C/mRNA + laser (G4), PSB + laser (G5), PSB@Nb_1.33_C + laser (G6), PSB@Nb_1.33_C/mRNA + laser (G7). *p <0.05, **p <0.01, ***p <0.001, ****p <0.0001.

Based on the favorable effectiveness of the nanosystem in treating primary and distant tumors in vivo. The specific immune stimulation by PSB@Nb_1.33_C/mRNA nanovaccines was explored next. the CAF and TAM in tumor tissue were respectively characterized as α‐SMA positive and F4/80 positive cells to determine the translation of GFP‐mRNA by the delivery system (**Figure** **6**A). The results showed that under the 808 nm NIR laser, the GFP mRNA vaccine can translated in the CAF and TAM cells, with the mean fluorescence overlap coefficient of 0.21 and 0.19 respectively (Figure S24A, Supporting Information). The Ki‐67 protein is a cellular marker for cell proliferation. Based on this, we used the Ki‐67 positive area to explore the translation of GFP‐mRNA in tumor tissue (Figure 6B). The results showed that some green fluorescent protein expression was co‐located with the cells which are Ki‐67 positive, with a mean overlap coefficient of 0.38. Besides, we used the Ki‐67‐labeled tumor cells to explore the location of GFP‐mRNA in tumor tissue after different treatments (Figure S24B, Supporting Information). The results showed that GFP mRNA vaccine can be more co‐located with the tumor cells through the designed PSB@Nb_1.33_C/mRNA nanovaccine delivery system under the 808 nm NIR laser. To investigate the efficiency of WT1 editing in vivo, the WT1 protein and *WT1* mRNA were evaluated by employing western blot and RT‐qPCR (Figure 6C–E). The results showed that WT1 protein and *WT1* mRNA expression levels were elevated after treatment with PSB@Nb_1.33_C/mRNA + laser in vivo. Indicating that the designed nanovaccine delivery system efficiently mediated the upregulation of WT1 expression. Stimulation and expansion of antigen‐specific CD8^+^ T cell populations are crucial for cancer immunotherapy. We carried out experiments to evaluate the WT1‐specific immunization efficacy of PSB@Nb_1.33_C/mRNA nanovaccines in vivo. mRNA vaccines show optimal properties for the activation of CTL responses, the PSB@Nb_1.33_C/mRNA induced WT1‐specific T cells immune responses were investigated to explore the enrichment of WT1‐specific CTLs within the tumor (Figure 6F,G). Notably, stronger WT1‐specific T‐cell mediated immune responses occur in the group of PSB@Nb_1.33_C/mRNA under the 808 nm NIR laser, which indicates that the delivery system resulted in the mRNA vaccine translation, and then the antigen can trigger WT1‐specific anti‐tumor immune response was generated in mice. Furthermore, the model protein antigen ovalbumin (OVA) mRNA was used to demonstrate the DCs present in the mRNA vaccine through the designed efficient delivery system of mRNA vaccine (Figure S25, Supporting Information). The results showed that PSB@Nb_1.33_C/mRNA triggered the highest level of CD11c^+^SIINFEKL‐H‐2Kb^+^ antigen cross‐presented DCs in draining lymph nodes.

### 2.6 Anti‐Tumor Immune Mechanisms In Vivo

**Figure 7:**
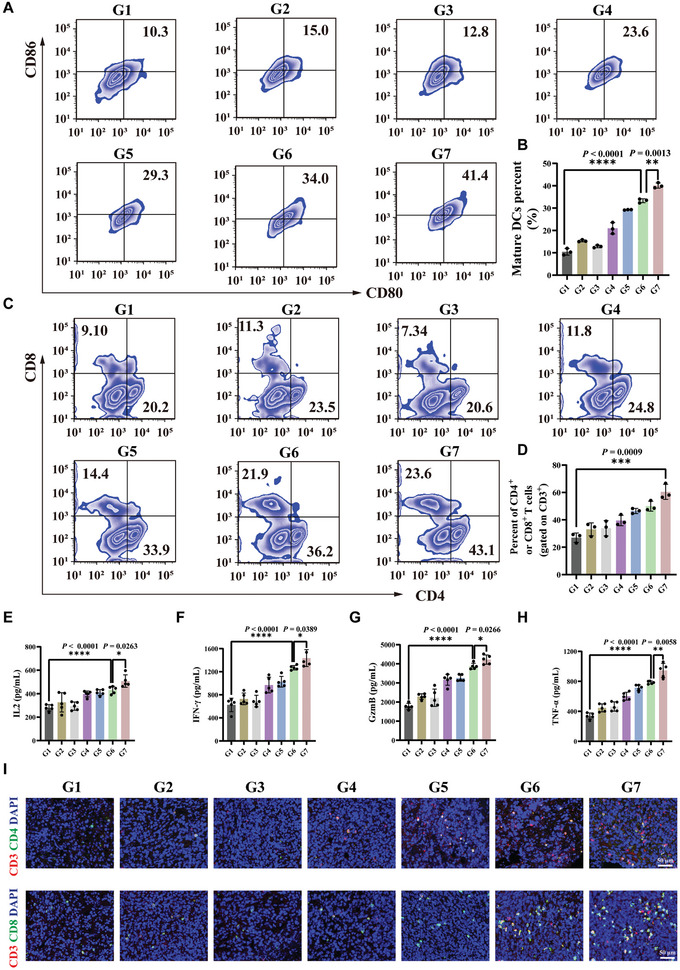
Immune mechanism in vivo. A) Typical FCM showing DC maturation and B) quantitative analysis of 4T1 tumor tissue following the various initial treatments (*n* = 3). C) Typical FCM and D) quantitative analysis of the CD4^+^ and CD8^+^ T cells in the primary 4T1 tumor tissue following the various initial treatments (*n* = 3). E–H) Levels of IFN‐γ, TNF‐α, IL‐2, and GzmB in primary tumor tissues following the various initial treatments (*n* = 5). I) Immunofluorescence images depicting the proliferation of CD3^+^ CD4^+^ and CD3^+^ CD8^+^ CTLs in the primary tumor tissue sections after various treatments. Control (G1), PSB@Nb_1.33_C/mRNA (G2), laser (G3), Nb_1.33_C/mRNA + laser (G4), PSB + laser (G5), PSB@Nb_1.33_C + laser (G6), and PSB@Nb_1.33_C/mRNA + laser (G7). *p <0.05, **p <0.01, ***p <0.001, ****p <0.0001.

The immune cell response in the tumor and lymph node tissues was evaluated to further assess the underlying anti‐tumor mechanisms of the PSB@Nb_1.33_C/mRNA nanosystem after NIR laser irradiation. DC maturation was evaluated to investigate their ability to activate innate and adaptive immunity in vivo. The typical flow cytometric analysis of DC maturation and quantitative analysis indicated that the relative ratios of mature DCs in the PSB@Nb_1.33_C/mRNA + laser group were significantly higher than those in the other groups, with an average of 40.1% (**Figure** **7**A,B). In addition, the results of FCM showed highly significant infiltration of CD4^+^ and CD8^+^ T cells in the primary tumor tissue of the PSB@Nb_1.33_C/mRNA nanosystem after NIR laser irradiation, which was 2.56‐fold more than that of the control group (Figure 7C,D). Furthermore, analysis of the regulatory CD3^+^ CD4^+^ Foxp3^+^ cells in the tumors showed that the accumulation of Tregs was significantly downregulated in the PSB@Nb_1.33_C/mRNA + laser group (Figure S26A,B, Supporting Information). This indicated that the nanosystem can improve the anti‐tumor effect by relieving immunosuppression. In addition, the ratio of M1‐ and M2‐like macrophages was determined, which showed that the macrophage phenotype significantly transitioned from the M2 to the M1 subphenotype in the PSB@Nb_1.33_C/mRNA + laser group, indicating that the nanosystem was beneficial for killing the tumor cells (Figure S26C,D, Supporting Information). Furthermore, T cells in the lymph nodes indicated the activation of the immune system. FCM analysis revealed that the infiltration of CD4^+^ and CD8^+^ T cells in the PSB@Nb_1.33_C/mRNA + laser group was higher than that in the control group. The CD4^+^ and CD8^+^ T cells of the lymph nodes were investigated using FCM to confirm immune system activation more rigorously (Figure S27A,B, Supporting Information). Similarly, the designed treatment strategy increased the number of CD4^+^ and CD8^+^ T cells in the lymph nodes in vivo. The analysis of flow cytometry gating strategy was also shown (Figures S28–S30, Supporting Information). During the execution of these anti‐tumor immune processes, the levels of certain typical pro‐inflammatory cytokines, namely, IL 2, TNF‐α, IFN‐γ, and granzyme B (GzmB), were analyzed to assess the activation of the immune system. The levels of these cytokines were considerably upregulated in the PSB@Nb_1.33_C/mRNA + laser group (Figure 7E–H). The area positive for CD4^+^ and CD8^+^ T cells in the immunofluorescence images of the primary tumor tissue slices was quantified (Figure 7I; Figure S31A–D, Supporting Information). The results suggested that the positive area increased significantly with the PSB@Nb_1.33_C/mRNA delivery system after 808 nm NIR laser irradiation compared to those observed with the other treatments. Thus, the designed mRNA vaccine delivery platform can activate the immune system. In addition, CD4^+^ and CD8^+^ T cells were detected in distant tumor tissues. Once the first treatment for the primary tumor was administered, the distant tumor tissues were removed, and immunofluorescence staining was performed (Figure S32A–D, Supporting Information). The results suggested that the area positive for the CD4^+^ and CD8^+^ T cells increased significantly in the PSB@Nb_1.33_C/mRNA + laser treatment group compared to those in the other groups, indicating that the designed strategy activated the immune system to attack distant tumors. The PSB@Nb_1.33_C/mRNA delivery nanosystem targeted the 4T1 tumor cells and caused ICD via PTT under NIR irradiation. Thus, the production of DAMPs, including CRT, HSP70, and HMGB1, promoted DC maturation. The delivery system precisely and efficiently targeted tumor cells for *WT1* mRNA vaccine delivery. After its release from the nanomaterials, the mRNA can be internalized, transported to the cytosol, and then read by ribosomes. This translated into the protein that upregulated the expression of tumor‐associated antigens. The DAMPs and upregulation of WT1 facilitated DC activation, and mature DCs presented these antigens to T cells to trigger a robust tumor‐specific T cell response. The stimulation of anti‐tumor immune activity was responsible for killing tumor cells in vivo.

### 2.7 Long‐Term Immune Effects On Lung Metastases In Vivo

**Figure 8:**
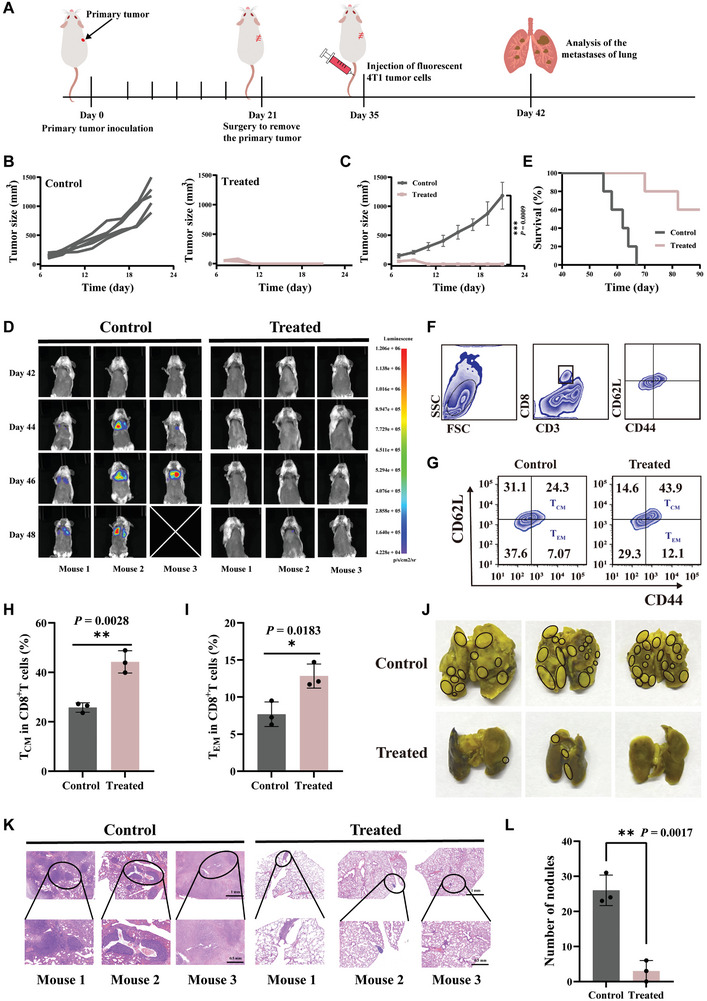
Effectiveness of the vaccine against lung metastases. A) Diagram outlining the progression and therapeutic procedure of lung metastasis. B) The primary tumor growth curves in different groups *(n* = 5). C) Average tumor growth curves of the primary tumor (*n* = 5). D) In vivo bioluminescence images to track the outgrowth of 4T1 tumor cells after different treatments. E) Survival curves of 4T1 tumor‐bearing mice after different treatments (Control, Treated) (*n* = 5). *p <0.05, **p <0.01, ***p <0.001, ****p <0.0001. F) Gating strategies for isolating T cells (CD3^+^ CD8^+^ CD44^+^ CD62L^+^) (Tcm) and (CD3^+^ CD8^+^ CD44^+^ CD62L^−^) (Tem). G) Typical FCM and statistical data of central memory T cells (Tcm) H) and effector memory T cells (Tem) I) in the spleen tissue (gated on CD3^+^CD8^+^ T cells) after 24 h of the first treatment (*n* = 3). J) Representative photographs of the number of lung metastatic nodules in different groups (Control, Treated) (*n* = 3). K) Representative hematoxylin and eosin (H&E) staining analysis of the lung tissue. L) The counts of the number of lung metastatic nodules in different groups (Control, Treated) (*n* = 3). *p < 0.05, **p < 0.01, ***p < 0.001, ****p < 0.0001.

As mentioned, the PSB@Nb_1.33_C/mRNA nanosystem exhibited outstanding therapeutic efficacy in primary and distant tumors. Considering the pivotal importance of immune memory in sustaining long‐term anti‐tumor benefits, its ability to counter lung metastasis was investigated. A model of lung metastasis was established to analyze the anti‐pulmonary metastatic effects of PSB@Nb_1.33_C/mRNA (**Figure** **8**A). Mice were randomly allocated into two groups after the 4T1 tumor cells were injected and sustained growth was observed for 7 days, namely, those that did not receive any treatment (Control) and those that received PSB@Nb_1.33_C/mRNA + laser (Treated). First, the primary tumor volume was monitored and recorded after every 2 days. The results indicated that PSB@Nb_1.33_C/mRNA + laser significantly inhibited tumor growth compared to that in the control group (Figure 8B,C). The tumors were surgically removed at the end of the recording period in the control group. After eliminating the primary tumor in the control group, the mice were intravenously injected with fluorescently labeled 4T1 cells. Gradually increasing bioluminescence signals were detected over time in the control and treated mice. These results showed that mice treated with PSB@Nb_1.33_C/mRNA under NIR irradiation exhibited a feeble fluorescence signal compared to that in the control mice, suggesting that diffuse lung metastasis could be suppressed to a large extent (Figure 8D). The mice in the treated group survived longer (Figure 8E). To further verify the associated immune cell responses induced by mRNA vaccination‐based combination therapy, spleen tissue was collected to estimate the number of memory CD8^+^ T cells. The mice of the treated group harbored significantly higher levels of central (CD62L^+^ CD44^+^) and effector (CD62L^−^ CD44^+^) memory T cells than that in the control group (Figure 8F–I). Representative metastatic lung nodules were recorded using both frontal and back photographs (Figure 8J; Figure S33, Supporting Information). In addition, HE staining was performed to further verify the suppression of lung metastases in the PSB@Nb_1.33_C/mRNA nanosystem (Figure 8K). The nanosystem significantly reduced the number of nodules in the lungs compared to that in the untreated group (Figure 8L).

## 3 Discussion

mRNA vaccines have remarkable potential for cancer therapy due to their safety, efficacy, and ease of industrial production.^[^ ^21^ ^]^ Vehicle‐loaded mRNA vaccines efficiently express tumor antigens in APCs, facilitating APC activation and innate/adaptive immune stimulation during vaccination.^[^ ^22^ ^]^ One of the most important aspects of how cancer vaccines activate the immune system to be more effective in effectively attacking cancer cells is what cancer antigen is used. Wild‐type *WT1* is expressed in various cancers. Therefore, the *WT1* mRNA and protein may act as promising targets for cancer therapy. It is one of the most well‐characterized and immunogenic TAAs and is an ideal target for therapeutic cancer vaccines. As a tumor‐associated antigen, high levels of WT1 protein expression can induce an immune response. Therefore, upregulating the expression of WT1 protein may be an effective strategy for treating breast cancer. However, the delivery of mRNA vaccines constitutes a major bottleneck in tumor treatment. Lipid NPs are major mRNA carriers used for delivery in clinical settings. Nevertheless, this encapsulation strategy is unsuitable for delivering personalized tumor vaccines because of the complexity and heterogeneity of tumor antigens.^[^ ^23^ ^]^

In this study, we designed and synthesized Nb_1.33_C/mRNA and used the system to deliver the mRNA vaccine in tumor tissue efficiently and safely. In addition, PSB effectively targeted the tumor site as an excellent delivery carrier via hypoxia targeting and directly attacked tumor tissue after NIR laser irradiation via PTT. The designed PSB@Nb_1.33_C/mRNA delivery nanosystem precisely targeted 4T1 tumor cells and caused ICD via PTT after NIR irradiation. The production of DAMPs, including CRT, HSP70, and HMGB1, promoted DC maturation. The delivery system accurately and efficiently targeted tumor cells for *WT1* mRNA vaccine delivery. After being released from the nanomaterials, the mRNA was internalized, transported to the cytosol, and then read by ribosomes. This was translated into a protein that upregulated the expression of TAAs. The expression of DAMPs and upregulation of the WT1 protein considerably facilitated DC activation, and the mature DCs presented these antigens to T cells to induce a strong tumor‐specific T cell response. Stimulating anti‐tumor immune effects resulted in the killing of tumor cells in vivo. The nanosystem killed 4T1 tumor cells via PTT under NIR laser irradiation in vitro and delivered the *WT1* mRNA encoding tumor‐associated antigens to APCs within the tumor in vivo. This provides a potent and powerful combination therapy for treating tumors.

This study showed that tumor tissues can be effectively attacked via the PTT property of PSB and 2D *i*MXene nanosheets (Nb_1.33_C). A novel delivery technology for introducing personalized mRNA vaccines in tumors was developed. The system successfully delivered an mRNA vaccine and upregulated WT1 protein expression. The therapeutic effects indicated that the designed delivery vehicle robustly activated the immune system to damage 4T1 tumor tissues and inhibited tumor metastasis in mice. Furthermore, the expansion of effective immune memory cells produced by the nanosystem can counter 4T1 tumor rechallenge.

In summary, this study provides a novel delivery nanocarrier for mRNA vaccines and a combination strategy for treating tumors, paving the way for its wide‐ranging application in personalized tumor vaccines, other vaccine applications, and cancer therapy. Furthermore, our innovative approach has remarkable potential in the field of mRNA nanovaccines to achieve photo‐immunogene cancer therapy and general applications in the treatment of other tumors.

## 4 Experimental Section

### Characterization

TEM (FEI Talos F200X) was used to observe the morphology and element mapping of the delivery nanosystem. SEM and the corresponding element mapping were performed using S‐4800 (Hitachi Company). AFM was performed using a Bruker Multimode 8‐HR instrument. Dynamic light scattering (DLS) and zeta potential measurements were performed using a Nano ZS 960 Malvern Zetasizer Nanoseries instrument (Malvern Instruments Ltd., UK). NETZSCH STA 449 F3 Jupiter thermal analyzer was used to explore the thermogravimetric analysis. The UV‐vis spectra were collected using a UV‐1900i Shimadzu spectroscope. Temperature was recorded using the FLIR A325SC camera and an infrared thermal imaging device. Photothermal performance was measured and analyzed using an 808 nm NIR laser (Shanghai Connect Fiber Optics Company). A CLSM (ZEISS LSM900) was used to acquire confocal images. FCM was performed using a flow cytometer (BD LSR Fortessa).

### Fabrication of mPEG‐PEI‐Coated Nb_1.33_C

Synthesis of (Nb_2/3_Y_1/3_)_2_AlC powders: for synthesizing the (Nb_2/3_Y_1/3_)_2_AlC MAX‐phase ceramics, Nb, Y, Al, and C powders were mixed in 4/3:2/3:1:1 molar ratio. Subsequently, the resulting hybrid powders were compacted into discs using cold pressing and then heated in an argon (Ar) atmosphere at 1450 °C, with the temperature increasing at the rate of 8 °C min^−1^ over 2 h. After cooling to room temperature, the samples were delicately crushed to obtain the final (Nb_2/3_Y_1/3_)_2_AlC *i*‐MAX product.

Synthesis of Nb_1.33_C nanosheets (*i*MXenes): The (Nb_2/3_Y_1/3_)_2_AlC powder (2 g) was carefully added to a Teflon bottle, which already contained 20 mL HF (Macklin, China). The bottle was then placed on a stirring plate at room temperature and stirred for 30 h using a Teflon‐coated magnet. After completion of the etching procedure, the generated products were separated via centrifugation. The resulting suspension was washed several times with degassed water until the pH reached ≈ 6. Within each washing cycle, the centrifuge tube was filled with degassed water (30 mL) and agitated for 10 min. Subsequently, centrifugation was performed at 5000 rpm for 1 min. An aqueous solution (5 mL) of 54−56 wt.% TBAOH ((C_4_H_9_)_4_NOH [Sigma Aldrich, Sweden]) was prepared, and the settled powder (1 g) was introduced into the solution. The mixture was manually shaken for 15 min and then subjected to three wash cycles, with 35 mL degassed water per cycle. The mixture was centrifuged for 1 h at 6000 rpm and the supernatant was collected. A nanoporous polypropylene membrane was used to filter the suspensions. A single‐layered and flexible Nb_1.33_C nanosheet was obtained from the membrane.

mPEG‐CHO was synthesized using an approach described in a previously published study.^[^ ^24^ ^]^ mPEG‐d‐PEI was formed by mixing PEI and mPEG‐CHO in a 2:1 weight ratio at pH 7.4 for 30 min. Nb_1.33_C was then added to the resulting solution to prepare the mPEG‐d‐PEI‐coated Nb_1.33_C. mPEG‐PEI‐coated Nb_1.33_C and *WT1* mRNA were mixed and incubated for 30 min to obtain Nb_1.33_C/mRNA.

In the exponential growth phase, PSB was centrifuged (6000 rpm, 7 min) and dispersed in phosphate‐buffered saline (PBS) to wash the bacteria. The Nb_1.33_C/mRNA solution was added dropwise to the activated PSB suspension with stirring at room temperature for 30 min to obtain the PSB@ Nb_1.33_C/mRNA delivery nanosystem.

### Analysis of ICD In Vitro and WT1 Protein Expression Assay

Following 24 h of various treatments (Control, PSB@Nb_1.33_C/mRNA, laser, Nb_1.33_C/mRNA + laser, PSB + laser, PSB@Nb_1.33_C + laser, and PSB@Nb_1.33_C/mRNA + laser), 4T1 cells in 6‐well plates (5 × 10^5^) were washed twice with PBS, after which the cells were placed on ice for 30 min, collected, and carefully inverted to ensure proper mixing. Following centrifugation at 12 000 rpm, the bicinchoninic acid protein assay kit was used to measure the total protein concentration in the resulting supernatant.

Thirty micrograms of total protein were analyzed using sodium dodecyl sulfate‐polyacrylamide gel electrophoresis (SDS‐PAGE) on a 10% gel and subsequently transferred onto a polyvinylidene fluoride membrane. The samples were treated with QuickBlock blocking buffer for 15 min at room temperature to prevent non‐specific binding. Subsequently, the membranes were incubated for 12 h in solutions containing antibodies against HSP70 (Affinity, AF5466), HMGB1 (Affinity, AF7020), WT1 (Proteintech, 12609‐1‐AP), CRT (Affinity, DF6211), GAPDH (Affinity, AF7021), and a rabbit polyclonal antibody, followed by treatment with a conjugated secondary antibody (Beyotime) for 1 h at room temperature. The membranes were washed thrice for 10 min each using 0.02% Tris‐buffered saline and Tween 20 buffer. Pierce ECL western blotting substrate was applied to visualize the target protein. To ascertain the ICD induced by the nanosystem, the surface expression of CRT and release of HMGB1 and HSP70 were assessed using immunofluorescence in vitro. 4T1 tumor cells were seeded in CLSM‐specific culture dishes at the density of 1 × 10^5^ cells per well and incubated under the following conditions: Control, PSB@Nb_1.33_C/mRNA, laser, Nb_1.33_C/mRNA + laser, PSB + laser, PSB@Nb_1.33_C + laser, and PSB@Nb_1.33_C/mRNA + laser. Following 12 h of incubation, the cells were washed thrice with PBS and then exposed to rabbit monoclonal antibodies against HMGB1, HSP70, and CRT for 1 h, followed by staining with Alexa Fluor 488 or 555‐conjugated goat anti‐rabbit IgG for 1 h. 4′,6‐diamidino‐2‐phenylindole (DAPI) staining was performed for 20 min. The tumor cells were visualized using CLSM.

### PTT Performance of PSB

One hundred microliters of different concentrations (1 × 10^9^, 0.5 × 10^9^, 0.25 × 10^9^, 0.125 × 10^9^, 0.0625 × 10^9^ CFU mL^−1^) of PSB solutions in PBS was treated with laser (808 nm, 1 W cm^−2^, 4 min). Then, 100 µL PSB (10^9^ CFU mL^−1^) in PBS was treated with 808 nm laser (0.5, 0.75, 1, 1.25, 1.5 W cm^−2^). To investigate the potential impact of a series of irradiation treatments on the PTT property of PSB, PSB solutions (10^9^ CFU mL^−1^) were irradiated with an 808 nm NIR laser (1 W cm^−2^) for four cycles. A thermographic camera was used to capture and record the temperature measurements.

### PTT Performance of *i*MXene Nb_1.33_C

Various concentrations of 100 µL Nb_1.33_C (200, 100, 50, 25, 12.5, 0 µg mL^−1^) in PBS was treated with laser (808 nm, 1 W cm^−2^, 4 min). Then, 100 µL Nb_1.33_C (100 µg mL^−1^) in PBS was treated with different power densities of the NIP laser (808 nm, 0.5, 0.75, 1, 1.25, 1.5 W cm^−2^). Four cycles of NIR laser were applied to 100 µg mL^−1^ Nb_1.33_C samples to assess its potential effect on PTT efficiency. A thermographic camera was used to capture and record the temperature measurements.

### PTT Performance of the PSB@Nb_1.33_C Nanosystem

One hundred microliters of different concentrations of PSB@Nb_1.33_C in PBS were treated with laser (808 nm, 1 W cm^−2^, 4 min). Then, 100 µL PSB@Nb_1.33_C was treated with different power densities of the NIR laser (808 nm, 0.5, 0.75, 1, 1.25, 1.5 W cm^−2^). The PTT efficiency of the PSB@Nb_1.33_C solution and the effect of repeated treatments was assessed by subjecting the samples (PSB = 10^9^ CFU mL^−1^, Nb_1.33_C = 100 µg mL^−1^) to four cycles of 808 nm NIR laser exposure at the power density of 1 W cm^−2^. A thermographic camera was used to capture and record the temperature measurements.

### Agarose Gel Electrophoresis

A Nb_1.33_C delivery nanosystem was generated after co‐incubation of various mass ratios of Nb_1.33_ C/mRNA (0:1, 1:0, 0.5:1, 1:1, 2:1, 4:1, and 8:1). Following centrifugation, the supernatants were carefully loaded onto a 2% (w/v) agarose gel. Electrophoresis was performed at 80 V for 30 min. The samples were visualized using a UV illuminator using a Gel Doc System (Bio‐Rad).

### Cell Culture

The mouse‐derived 4T1 breast cancer cells were cultured at 37 °C in the presence of 5% CO_2_ in Dulbecco's modified Eagle's medium (Thermo Fisher Scientific) supplemented with 10% fetal bovine serum and 1% penicillin‐streptomycin.

### PSB Culture

PSB was cultured in an ATYP medium under anaerobic conditions. The liquid medium was sealed to maintain an anaerobic environment.

### Analysis of the Cellular Uptake and Lysosomal Escape of Nb_1.33_C/mRNA Nanoparticles

The distribution of mRNA within the cells was analyzed using CLSM (ZEISS LSM900) to examine the cellular structure of the Cy3‐labeled Nb_1.33_C/mRNA. The 4T1 cells were plated onto culture dishes specifically designed for CLSM at the density of 1 × 10^5^ cells for 12 h. Then, the Nb_1.33_C/mRNA nanosystem was introduced in the 4T1 cells and incubated (Nb_1.33_C = 100 µg mL^−1^) for different durations (0, 3, 6, 8, 10 12 h). DAPI and LysoTracker stained the nuclei blue and lysosome green, respectively.

### Evaluation of Cell Viability and Cellular Apoptosis

The in vitro therapeutic effects of PSB@Nb_1.33_C/mRNA were evaluated. 4T1 cells were seeded on 96‐well plates at the density of 10^4^ cells per well, followed by 12 h incubation at 37 °C in the presence of 5% CO_2_. Then, the cells were co‐incubated with different concentrations of PSB@Nb_1.33_C/mRNA (PSB = 10^9^ CFU mL^−1^, Nb_1.33_C = 0, 12.5, 25, 50, 100, 200, and 400 µg mL^−1^) for 8 h. Next, the cells in each well were washed thrice using PBS, and cell viability was measured using the standard CCK‐8 assay. The absorbance at 450 nm was measured for each well.

4T1 cells were seeded in the 96‐well plates (10^4^ cells per well) and cultured for 12 h (5% CO_2_, 37 °C) to investigate their therapeutic effects in vitro. Then, the cells were treated as follows: Control, PSB@Nb_1.33_C/mRNA, laser, Nb_1.33_C/mRNA + laser, PSB + laser, PSB@Nb_1.33_C + laser, PSB@Nb_1.33_C/mRNA + laser (PSB = 1 × 10^9^ CFU mL^−1^, Nb_1.33_C/mRNA = 100 µg mL^−1^, NIR = 1.0 W cm^−2^, 5 min). Next, each well was gently washed thrice with PBS to remove the residues. Cell viability was measured using a standard CCK‐8 assay. Absorbance at 450 nm was measured for each well.

Apoptosis was assessed using FCM. Six‐well plates were used for culturing 4T1 cells (10^5^ cells/well) for 8 h, during which the cells adhered to the bottom of the well and were then treated as described previously. Following trypsin‐mediated dissociation, the cells were gently washed with PBS to collect the cells. A combination of 5 µL PI and 10 µL fluorescein isothiocyanate (FITC) was added to the cells and incubated for 20 min in a dark environment. Apoptosis was quantified using FCM.

In addition, 1 × 10^5^ cells were seeded in every culture dish specially designed for CLSM to promote attachment to the culture plates during a 12‐h incubation period. After the treatments described above, a calcein‐AM)/PI solution was added to detect live and dead cells. A fluorescence microscope was used to visualize the cells.

### DC Maturation In Vitro

Eight‐week‐old female BALB/c mice were used to isolate bone marrow‐derived DCs (BMDCs) for analyzing DC maturation in vitro. First, in the transwell system, the DCs were seeded in the bottom chamber, while 4T1 tumor cells exposed to different pretreatment conditions (Control, PSB@Nb_1.33_C/mRNA, laser, Nb_1.33_C/mRNA + laser, PSB + laser, PSB@Nb_1.33_C + laser, and PSB@Nb_1.33_C/mRNA + laser) were placed on the upper part of the transwell and incubated for 24 h. DCs from different experimental groups were harvested and subjected to staining with anti‐CD80‐APC, anti‐CD86‐PECy7, and anti‐CD11c‐FITC antibodies. FCM was performed to evaluate the results. In addition, supernatants from the cell cultures were collected. The secretion of pro‐inflammatory cytokines, IFN‐γ, IL 2, and TNF‐α, by the DCs was assessed using respective ELISA kits.

### Real‐time PCR

qRT‐PCR was used to detect RNA expression in vitro. Total RNA was extracted using the Eastep total RNA extraction kit (Promega, Madison, WI, USA), following standard procedures. Next, the obtained total RNA was reverse transcribed into cDNA using the HiScript®II1st strand cDNA synthesis kit (Vazyme Biotech Co., Ltd., Nanjing, China). The SYBR Green PCR master mix (Biotechnology, Nanjing, China) was used for qRT‐PCR analysis. The expression data were standardized to *GAPDH* expression levels. All primers used in this study were purchased from Sangon Biotech (Shanghai, China). The primers used were as follows:

WT1 forward primer: 5′ CAGTTGTCAGAAAAAGTTTGCG 3′

WT1 reverse primer: 5′ GTGGGAGGAATTTCAAAATCAG 3′

### Hypoxia Targeting by PSB@Nb_1.33_C/mRNA

IR783‐labeled Nb_1.33_C/mRNA (Nb_1.33_C/mRNA = 10 mg kg^−1^) and IR783‐labeled PSB (PSB = 1 × 10^8^ CFU per mouse), and IR783‐labeled PSB@Nb_1.33_C/mRNA (PSB = 1 × 10^8^ CFU per mouse, Nb_1.33_C/mRNA = 10 mg kg^−1^) were intravenously injected into 4T1 tumor‐bearing female BALB/c mice when the tumor volume reached ≈200 mm^3^. The fluorescence distribution in the mice was monitored and captured using the VISQUE imaging system at various time points (0, 1, 2, 4, 8, 12, 24, and 48 h).

### Safety Evaluation of the PSB@Nb_1.33_C/mRNA Nanosystem In Vivo

Fifteen BALB/c mice were randomly assigned into five groups: Control, day 1, day 3, day 7, and day 30. Blood was collected after tail vein injection of PSB@Nb_1.33_C/mRNA (100 µL, PSB = 1 × 10^8^ CFU per mouse, Nb_1.33_C/mRNA = 10 mg kg^−1^). Liver and kidney function parameters and blood biochemical parameters were analyzed. HE staining was performed to examine the heart, liver, spleen, lung, and kidney tissues.

### Immune Response Against Primary Tumor of 4T1 tumor models

FCM was performed to isolate and examine different cell subtypes, including CD3^+^ CD4^+^ T cells, CD3^+^ CD8^+^ T cells, Tetramer^+^ CD8^+^ T cells, CD3^+^ CD4^+^ FOXP3^+^ T cells, CD11c^+^ CD86^+^ CD80^+^ DCs, SIINFEKL‐H‐2Kb^+^ CD11c^+^ DCs, CD45^+^ CD11b^+^ F4/80^+^CD86^+^ M1‐like macrophages, and CD45^+^ CD11b^+^ F4/80^+^ CD206^+^ M2‐like macrophages in tumor tissues. CD3^+^ CD4^+^ T cells and CD3^+^ CD8^+^ T cells were isolated from the lymph nodes and analyzed using FCM. ELISA kits were used to assess the level of inflammatory cytokines, such as TNF‐α, IFN‐γ, IL‐2, and GzmB, in the 4T1 primary tumors per the manufacturer's instructions. In addition, immunofluorescence staining was performed on the primary tumor tissue slices to visualize CD3^+^ CD4^+^ and CD3^+^ CD8^+^ CTL proliferation. Following the three treatments, the mice were euthanized, and immunofluorescence staining of primary tumor tissue slices was performed to analyze the presence of Ki‐67, HE, and TUNEL. After the first treatments, the mice were euthanized, and immunofluorescence staining of tumor tissue slices was performed to analyze the presence of GFP, CAF(α‐SMA positive), RAW 264.7 (F4/80 positive), and 4T1 (Ki‐67 positive).

### Immune Response Against Distant Tumor

Following the injection of the 4T1 tumors (1 × 10^6^ cells) into the left breast pad, the mice were maintained for 7 days for primary tumor development. In contrast, the contralateral breast pad was simultaneously injected with 1 × 10^6^ 4T1 tumor cells to establish distant tumor models. Mice were randomly allocated into seven groups, and the primary tumors were subjected to the previously described treatments. In contrast, no treatment was administered to distant tumors (*n* = 10). Regular measurements of tumor volumes (for both primary and distant tumors) were performed every 2 days for 7–21 days. Following the initial treatment, the mice were euthanized, and immunofluorescence staining of distal tumor tissue slices was performed to analyze the presence of proliferating CTLs expressing CD3^+^ CD4^+^ and CD3^+^ CD8^+^ markers.

### Assessment of Lung Metastasis

Lung metastatic tumor models were established using luc‐4T1 cells. 4T1 cells (1 × 10^6^) were injected into the right axillary region of mice and incubated for 7 days. The mice were grouped randomly into two groups and then treated using different strategies (control or not treated, PSB@Nb_1.33_C/mRNA + laser (treated) (PSB = 1 × 10^8^ CFU per mouse, Nb_1.33_C/mRNA = 10 mg kg^−1^, NIR = 1.0 W cm^−2^, 5 min). Tumor volumes were measured and recorded. Primary tumors in the control group were surgically removed after 21 days. The mice received tail vein injections of 1 × 10^5^ luc‐4T1 tumor cells. The presence of metastatic lung tumors was confirmed using a noninvasive bioluminescence imaging system based on luciferase, and the number of nodules was documented.

Examination of memory T cells involved collection of spleen tissues from the two groups of mice, followed by staining with anti‐CD8‐APC, anti‐CD3‐PerCP‐Cy5‐5, anti‐CD44‐PE, and anti‐CD62L‐PE‐Cy7 antibodies. Effector memory T cells (CD3^+^ CD8^+^ CD44^+^ CD62L^−^, Tem) and central memory T cells (CD3^+^ CD8^+^ CD44^+^ CD62L^+^, Tcm) were isolated and analyzed. Finally, HE staining was performed to visualize and record the presence of tumor nodules in the lung tissue.

### Statistical Analysis

All statistical analyses were executed using GraphPad Prism (version 9.0.0, GraphPad Software). One‐way analysis of variance (ANOVA) was used for multiple group comparisons. For other comparisons, an unpaired Student's t‐test was used when comparing two groups. Data represent means ± SD in each figure as indicated. The p‐value less than 0.05 was considered significant. *p < 0.05, **p < 0.01, ***p < 0.001, and ****p < 0.0001.

## Conflict of Interest

The authors declare that they have no conflict of interest.

## Author Contributions

S.Z., J.Y., and Y.L. contributed equally to this work. H.H.Y. W.W.Y. and H.X.X. designed the research strategy and experiments; S.Z., J.F.Y., Y.Y.L., B.X., Y.F., Y.L.Z. and S.Y.L. performed experiments and/or analyzed the data; H.H.Y., S.Z., J.F.Y., Y.K.S., L.F.W., L.P.S., X.H.X. and B.Y.Z. wrote the paper; H.H.Y., W.W.Y. and X.H.X. supervised the whole process.

## Supporting information

Supporting Information

## Data Availability

The data that support the findings of this study are available from the corresponding author upon reasonable request.;
