# Supplementary material for: Photosynthetic Bacteria‐Hitchhiking 2D iMXene‐mRNA Vaccine to Enable Photo‐Immunogene Cancer Therapy
Source: Adv Sci (Weinh). 2024 May 14;11(28):2307225. doi: 10.1002/advs.202307225 (PMC11267280; doi:10.1002/advs.202307225)
Supplement: Supplementary file 1 — Supporting Information [file ADVS-11-2307225-s001.pdf]

ADVANCED SCIENCE
Open Access

Supporting Information

for *Adv. Sci.*, DOI 10.1002/advs.202307225

Photosynthetic Bacteria-Hitchhiking 2D *i*MXene-mRNA Vaccine to Enable Photo-Immunogene Cancer Therapy

Shen Zhang, Jifeng Yu, Yunyun Liu, Bing Xiong, Yan Fang, Yuli Zhu, Shaoyue Li, Liping Sun, Boyang Zhou, Yikang Sun, Lifan Wang, Wenwen Yue, Haohao Yin* and Huixiong Xu**

Supporting Information

Photosynthetic bacteria-hitchhiking 2D *i*MXene-mRNA vaccine to enable photo-immunogene cancer therapy

Shen Zhang[1#], Jifeng Yu[1#], Yunyun Liu[2, 3, 4#], Bing Xiong[1], Yan Fang[2, 3, 4], Yuli Zhu[1], Shaoyue Li[2, 3, 4], Liping Sun[2, 3, 4], Boyang Zhou[1], Yikang Sun[1], Lifan Wang[1], Wenwen Yue[2, 3, 4*], Haohao Yin[1*] & Huixiong Xu[1*]

Correspondence to: xu.huixiong@zs-hospital.sh.cn; yin.haohao@zs-hospital.sh.cn; yuewen0902@163.com.

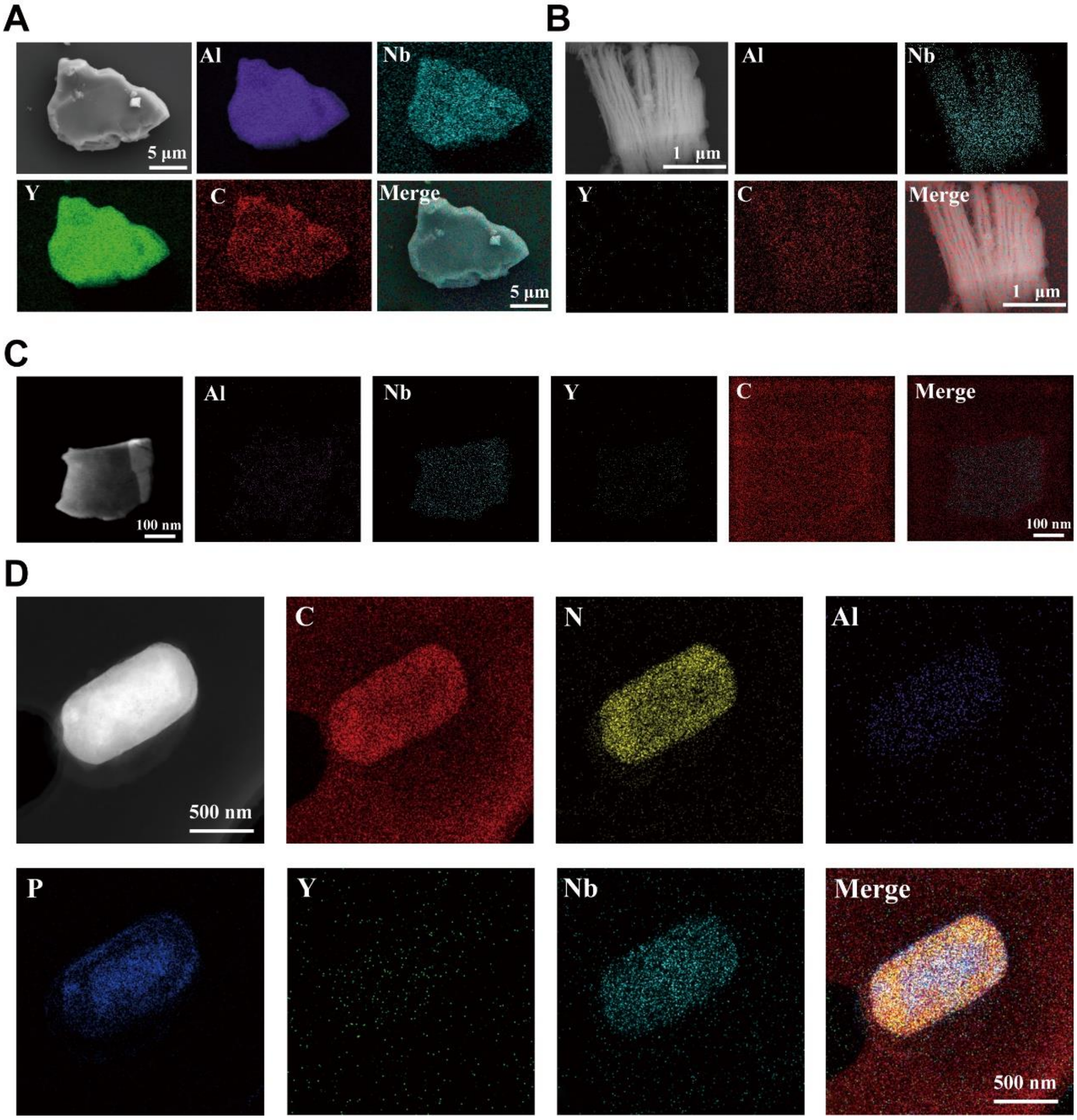

Figure S1. A) Corresponding elemental-mapping images (C, Al, Y and Nb elements) of $(Nb_{2/3}Y_{1/3})_2AlC$ before HF etching. B) Corresponding elemental-mapping images (C, Al, Y and Nb elements) of $(Nb_{2/3}Y_{1/3})_2AlC$ after HF etching. C) Corresponding elemental-mapping images (C, Al, Y and Nb elements) of $Nb_{1.33}C$ nanosheets. D) Corresponding elemental-mapping images (C, N, Al, P, Y and Nb elements) of PSB.

A

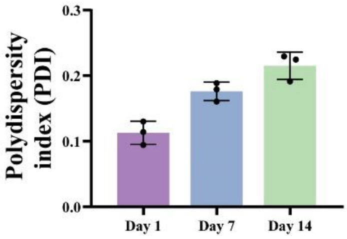

B

	Mean diameter (nm, mean ± SD)	Polydispersity index (mean ± SD)	Zeta potential (mV, mean ± SD)
Day 1	134.13 ± 1.20	0.11 ± 0.02	13.54 ± 1.42
Day 7	144.53 ± 2.06	0.18 ± 0.01	15.62 ± 0.49
Day 14	146.73 ± 1.31	0.23 ± 0.01	15.20 ± 0.13

Figure S2. A) The polydispersity index and B) DLS analysis of the $Nb_{1.33}C$/mRNA within 14 days.

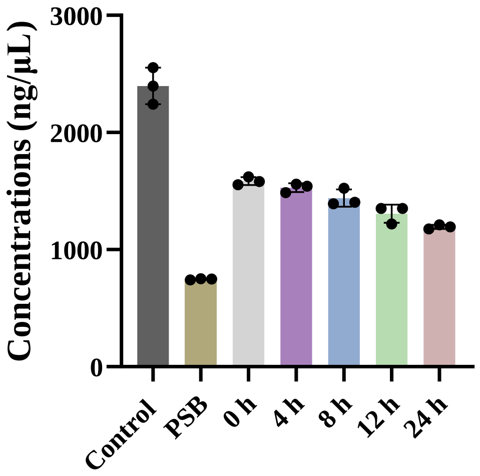

Figure S3. The RNA concentration of mRNA, PSB and PSB@$Nb_{1.33}C$/mRNA.

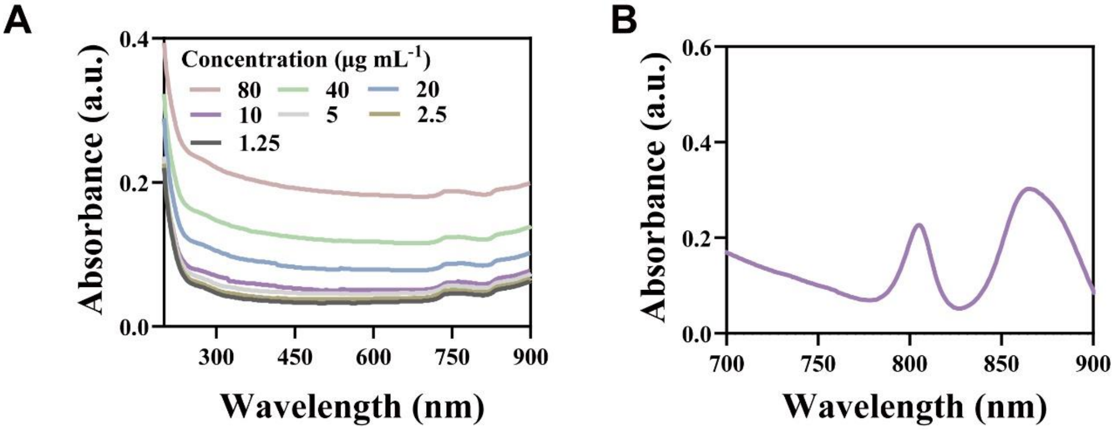

Figure S4. A) UV-vis absorption spectra of $Nb_{1.33}C$ in different concentration and B) PSB.

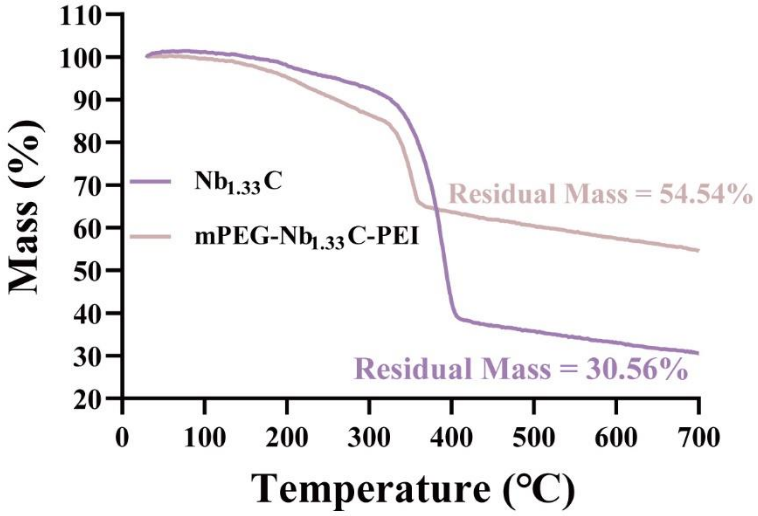

Figure S5. Thermogravimetric analysis of $Nb_{1.33}C$ and mPEG-$Nb_{1.33}C$-PEI.

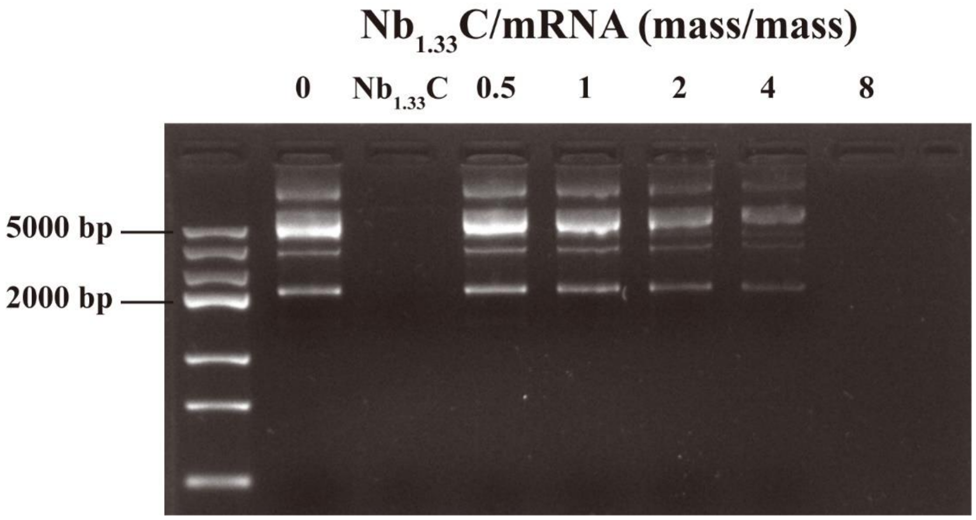

Figure S6. Agarose gel electrophoresis of different $Nb_{1.33}C$/mRNA ratios.

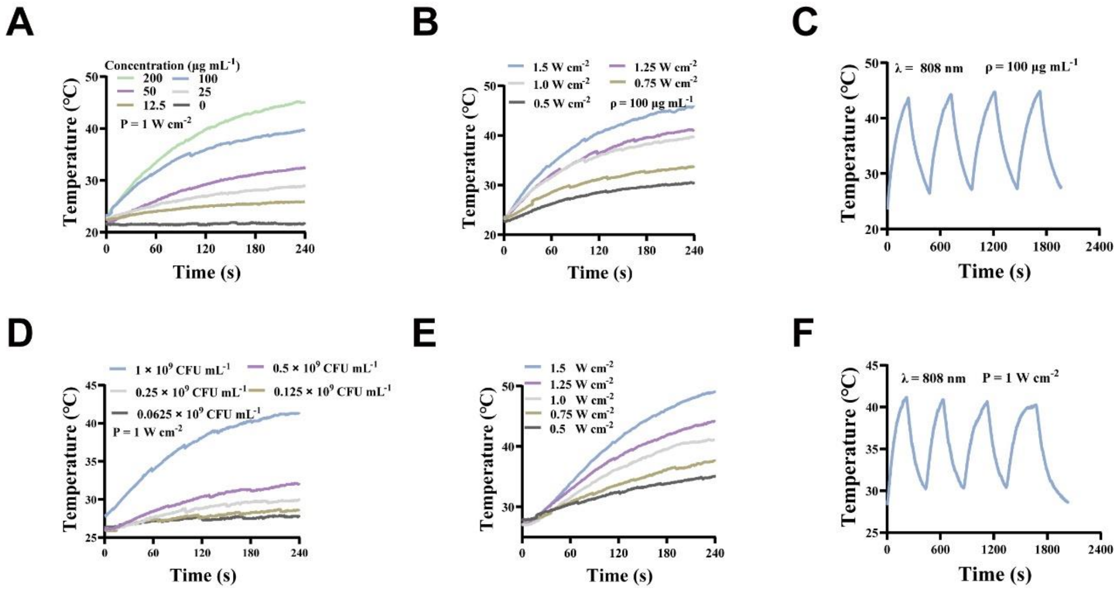

Figure S7. A) Photothermal temperature curves of $Nb_{1.33}C$ at different concentrations under an 808 nm laser with a power density of 1 W for 4 min. B) Photothermal heating curves of the $Nb_{1.33}C$ under various power intensities ($Nb_{1.33}C$ = 100 μg mL^{-1}). C) Temperature change in $Nb_{1.33}C$ ($Nb_{1.33}C$ = 100 μg mL^{-1}) under four irradiation/cooling cycles (1 W for 808 nm laser irradiation). D) Photothermal temperature curves of PSB at different concentrations under an 808 nm laser with a power density of 1 W for 4 min. E) Photothermal heating curves of the PSB (PSB = 10^9 CFU mL^{-1}) under various power intensities. F) Temperature change in PSB (PSB = 10^9 CFU mL^{-1}) under four irradiation/cooling cycles (1 W for 808 nm laser irradiation).

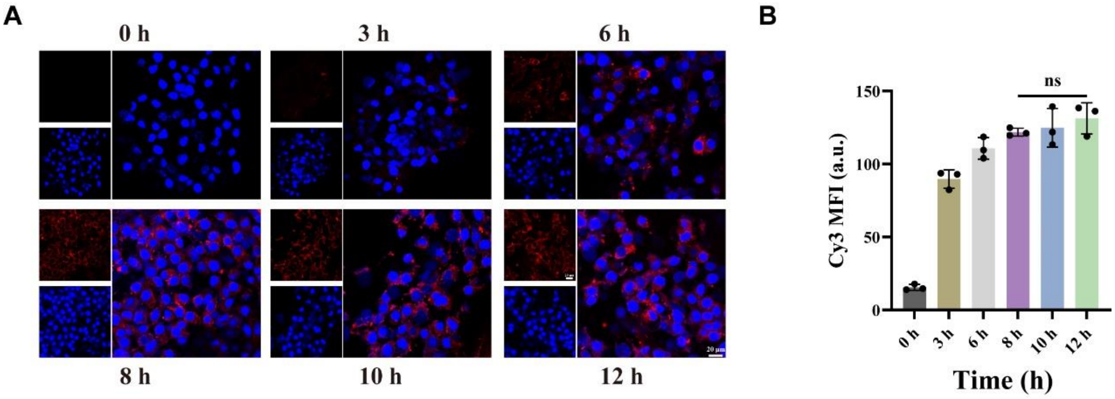

Figure S8. A) CLSM images and B) corresponding mean fluorescence intensity of Cy3-labeled $Nb_{1.33}C$/mRNA for different time points (*n* = 3).

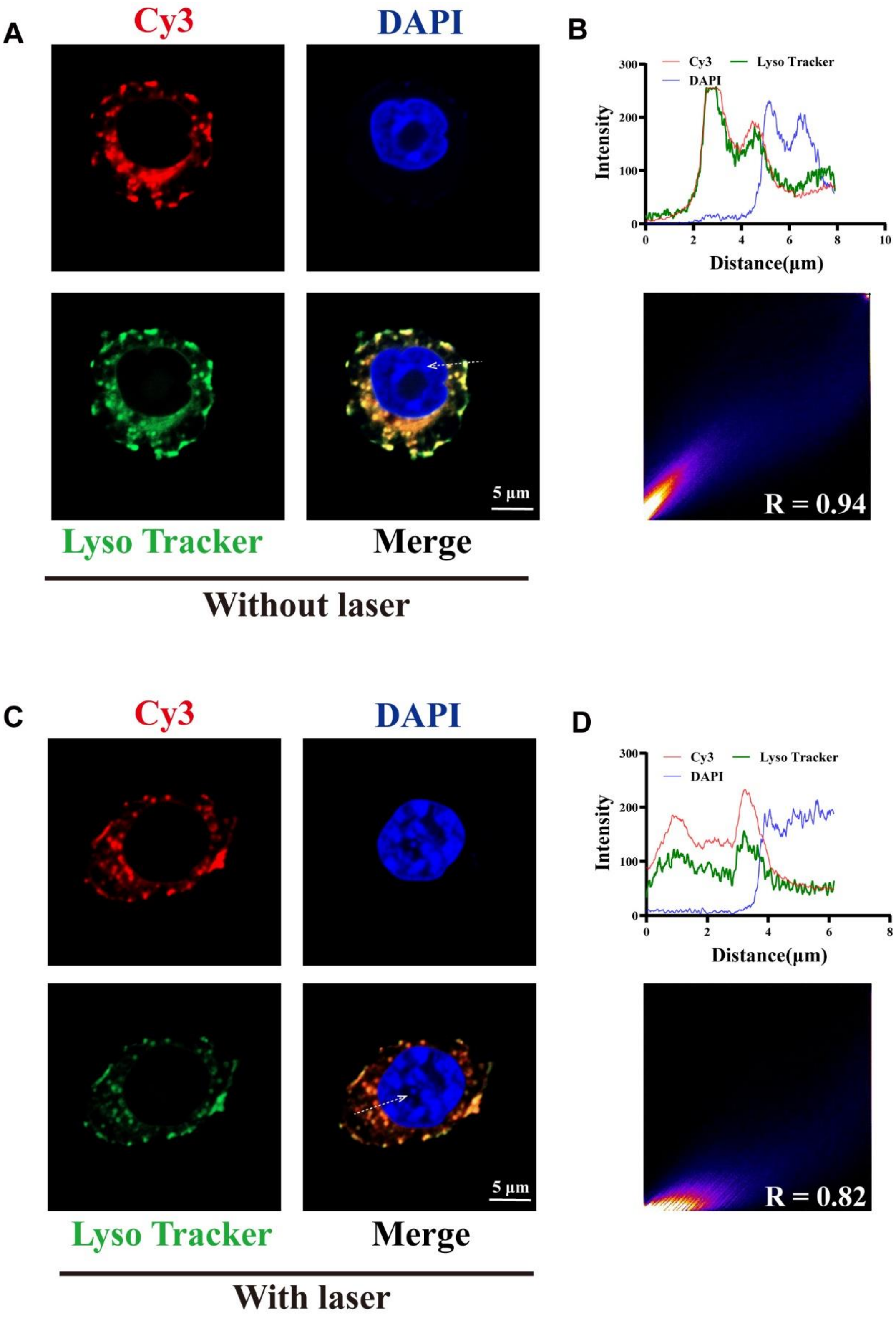

Figure S9. A) Typical CLSM images and B) corresponding fluorescence intensity and confocal imaging study of 4T1 cells co-staining with lysotracker Green and Cy3-labled $Nb_{1.33}C$/mRNA (without laser). C) Typical CLSM images and D) corresponding fluorescence intensity and confocal

imaging study of 4T1 cells co-staining with lysotracker Green and Cy3-labled $Nb_{1.33}C$/mRNA (with laser). R is the correlation coefficient.

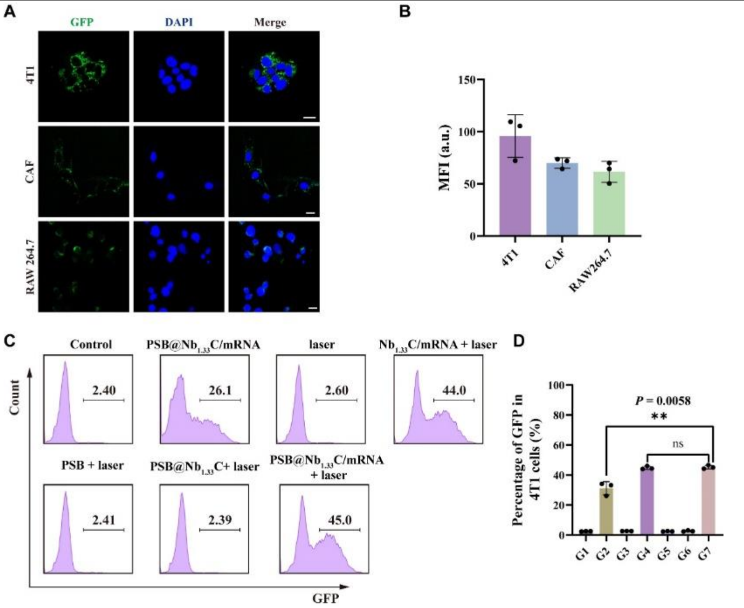

Figure S10. A) Typical CLSM images of GFP mRNA vaccine in 4T1, CAF and RAW 264.7 cells and B) the corresponding fluorescence intensity of GFP (*n* = 3). C) Typical FCM of GFP^+ 4T1 cells and D) quantitative analysis following the different treatments (*n* = 3).

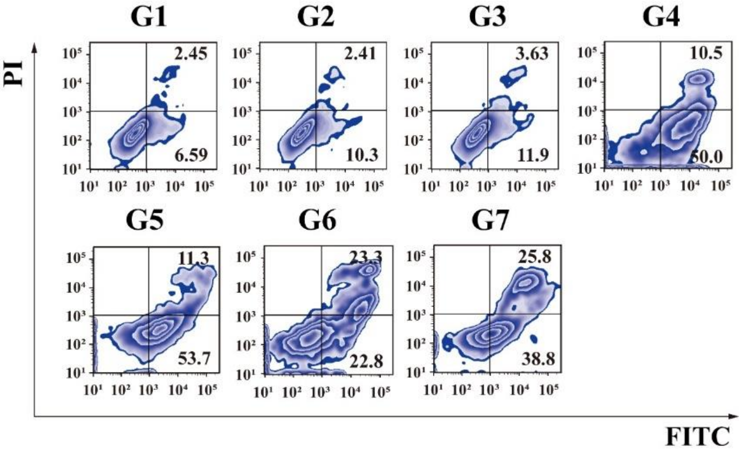

Figure S11. Flow cytometry apoptosis assay of 4T1 cells after different treatments which include Control (G1), PSB@$Nb_{1.33}C$/mRNA (G2), laser (G3), $Nb_{1.33}C$/mRNA + laser (G4), PSB + laser (G5), PSB@$Nb_{1.33}C$ + laser (G6), PSB@$Nb_{1.33}C$/mRNA + laser (G7) and stained with the annexin V-FITC and PI.

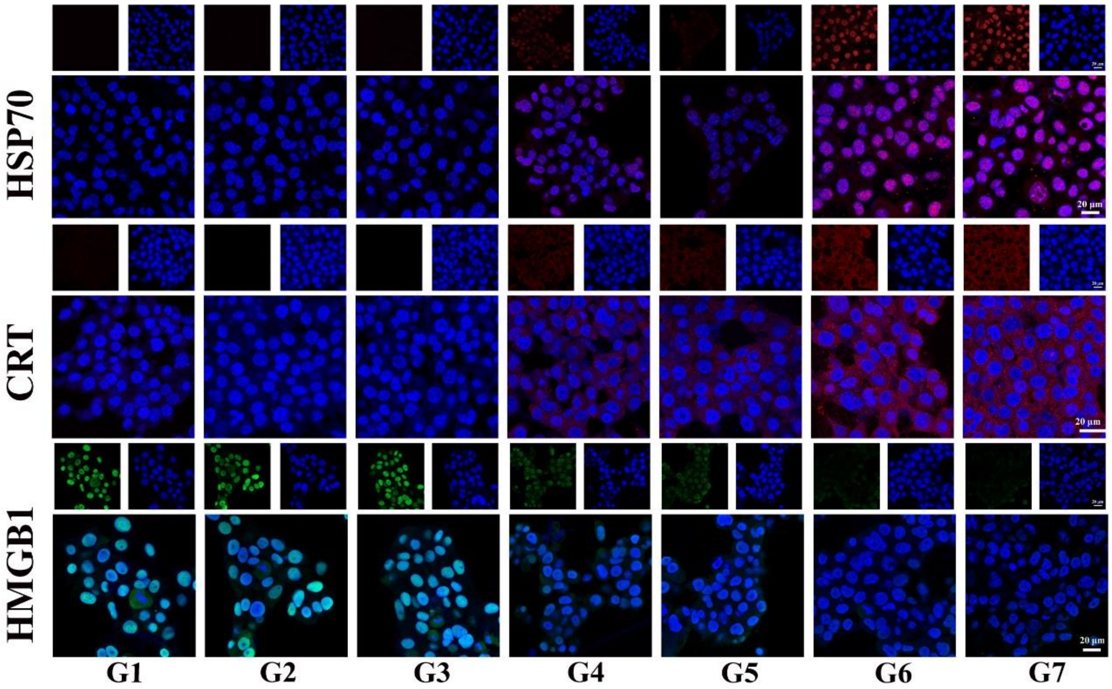

Figure S12. Individual and merge channels of Immunofluorescence analysis for HMGB1 (green), CRT (red) and HSP70 (red) (*n* = 3). Control (G1), PSB@$Nb_{1.33}C$/mRNA (G2), laser (G3), $Nb_{1.33}C$/mRNA + laser (G4), PSB + laser (G5), PSB@$Nb_{1.33}C$ + laser (G6), PSB@$Nb_{1.33}C$/mRNA + laser (G7).

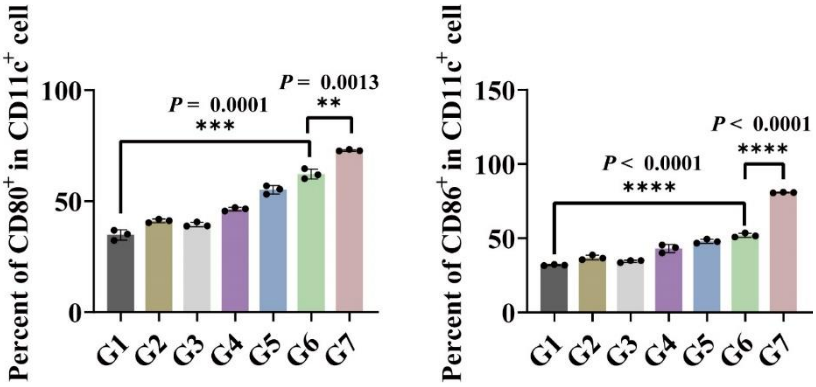

Figure S13. The statistical data of matured BMDCs ($CD80^+CD86^+CD11c^+$) after different treatments (n = 3). *P < 0.05, **P < 0.01, ***P < 0.001, ****P < 0.0001. Control (G1), PSB@$Nb_{1.33}C$/mRNA (G2), laser (G3), $Nb_{1.33}C$/mRNA + laser (G4), PSB + laser (G5), PSB@$Nb_{1.33}C$ + laser (G6), PSB@$Nb_{1.33}C$/mRNA + laser (G7).

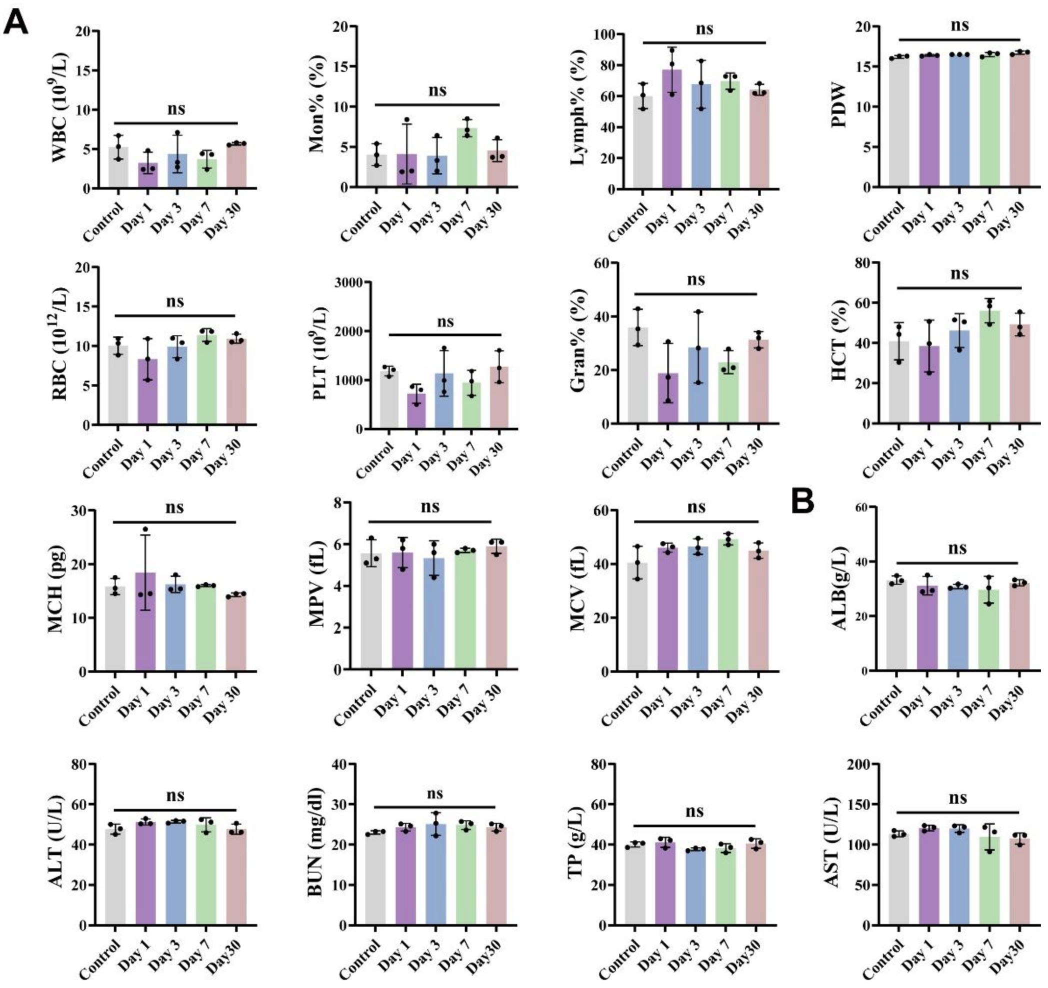

Figure S14. A) *In vivo* hematological indices. Hematological assays of mice at 1, 3, 7 and 30 days after PSB@$Nb_{1.33}C$/mRNA injection (*n* = 3). Control *i.e.* without any treatment. B) *In vivo* liver and kidney function index. Hematological assays of mice at 1, 3, 7 and 30 days after PSB@$Nb_{1.33}C$/mRNA injection (*n* = 3). Control *i.e.* without any treatment.

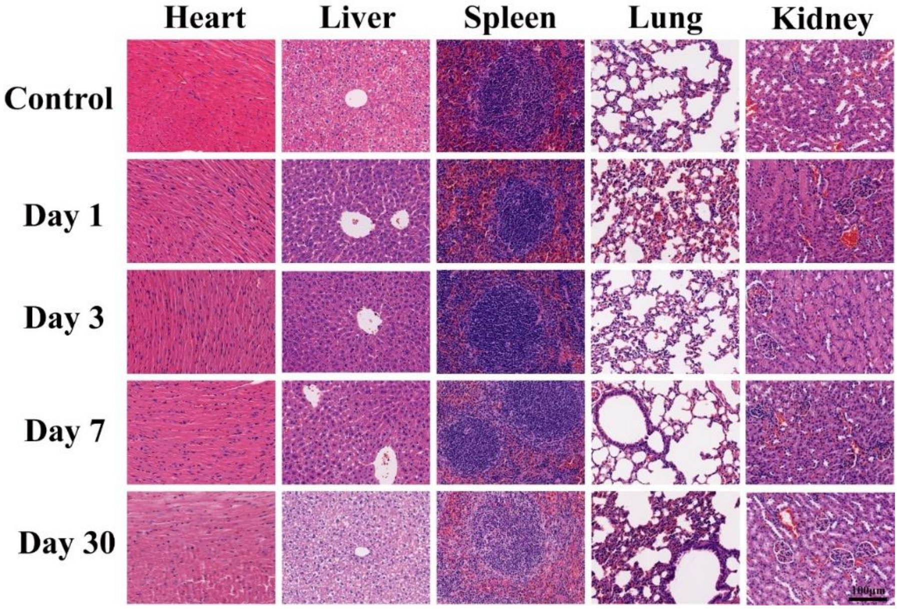

Figure S15. HE staining of histological sections of various organs in healthy mice after the PSB@$Nb_{1.33}C$/mRNA injection within one month (1, 3, 7 and 30 days), Control without any treatment.

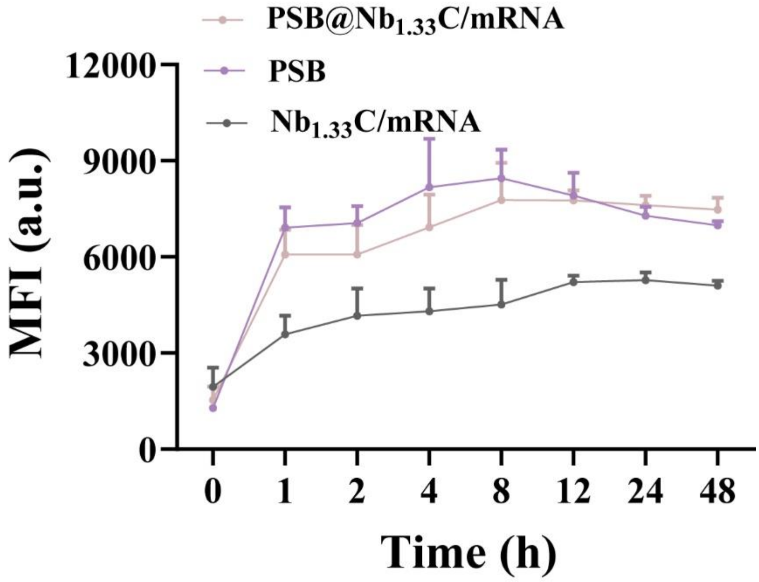

Figure S16. Corresponding fluorescence intensity of IR783-labeled $Nb_{1.33}C$/mRNA, PSB and PSB@$Nb_{1.33}C$/mRNA in mice, respectively. (1×10^8 CFU per mouse, $n = 3$). *$P < 0.05$, **$P < 0.01$, ***$P < 0.001$, ****$P < 0.0001$.

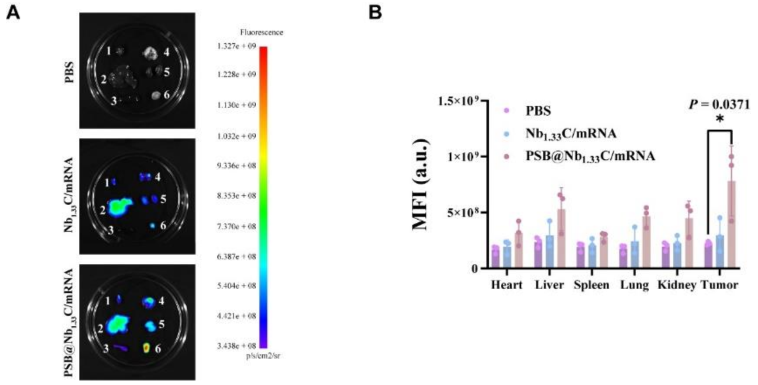

Figure S17. A) The fluorescence imaging and B) corresponding mean fluorescence intensity of the tumors and major organs (1. heart, 2. liver, 3. spleen, 4. lung ,5. kidney and 6. Tumor. n = 3 mice per group).

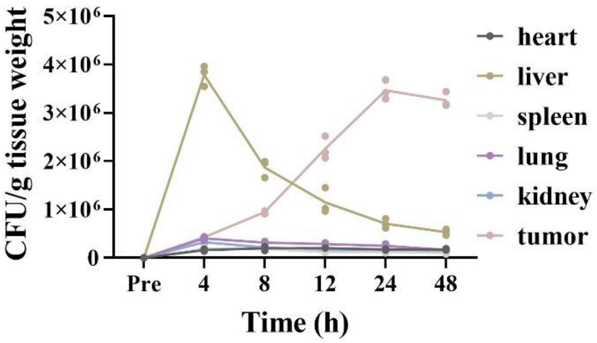

Figure S18. Corresponding quantitative analysis of bacterial colonization on the ATYP agar plates in various organs of 4T1-bearing mice in different time points after PSB injection (pre, 4, 8, 12, 24 and 48 h) (*n* = 3).

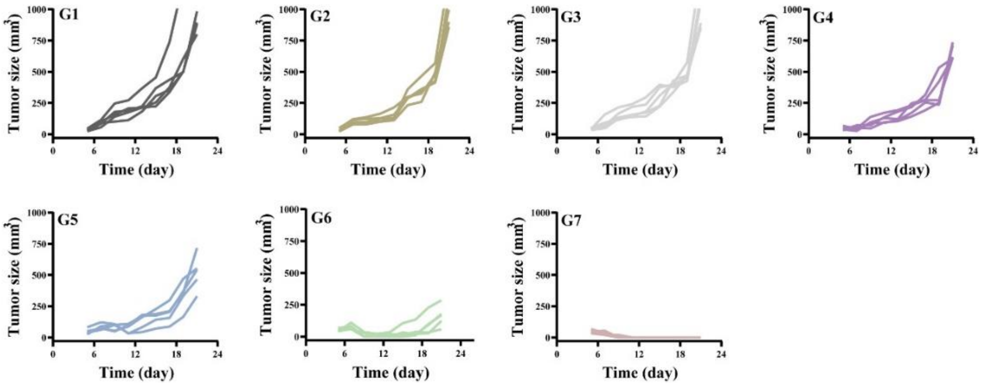

Figure S19. Individual primary tumor growth curves of 4T1 after being treated by Control (G1), PSB@$Nb_{1.33}C$/mRNA (G2), laser (G3), $Nb_{1.33}C$/mRNA + laser (G4), PSB + laser (G5), PSB@$Nb_{1.33}C$ + laser (G6), PSB@$Nb_{1.33}C$/mRNA + laser (G7) (n = 5).

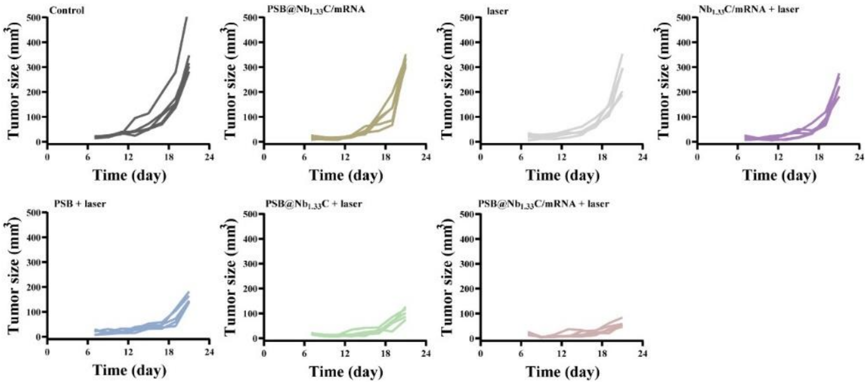

Figure S20. Individual distant tumor growth curves of 4T1 after being treated by Control, PSB@Nb$_{1.33}$C/mRNA, laser, Nb$_{1.33}$C/mRNA + laser, PSB + laser, PSB@Nb$_{1.33}$C + laser, PSB@Nb$_{1.33}$C/mRNA + laser (*n* = 5).

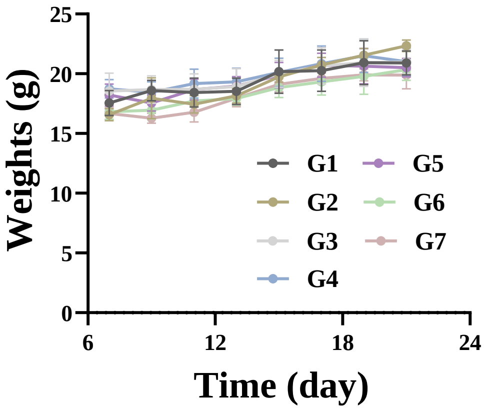

Figure S21. Body weight of the 4T1-tumor-bearing mice with different treatments (*n* = 5). Control (G1), PSB@$Nb_{1.33}C$/mRNA (G2), laser (G3), $Nb_{1.33}C$/mRNA + laser (G4), PSB + laser (G5), PSB@$Nb_{1.33}C$ + laser (G6), PSB@$Nb_{1.33}C$/mRNA + laser (G7) (*n* = 5).

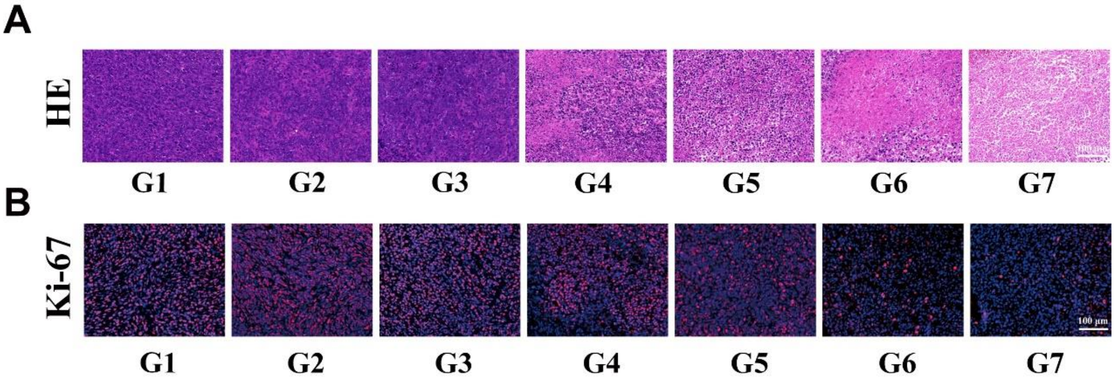

Figure S22. A) HE staining and B) Ki-67 images of 4T1 primary tumor tissue after different treatments. Control (G1), PSB@$Nb_{1.33}C$/mRNA (G2), laser (G3), $Nb_{1.33}C$/mRNA + laser (G4), PSB + laser (G5), PSB@$Nb_{1.33}C$ + laser (G6), PSB@$Nb_{1.33}C$/mRNA + laser (G7).

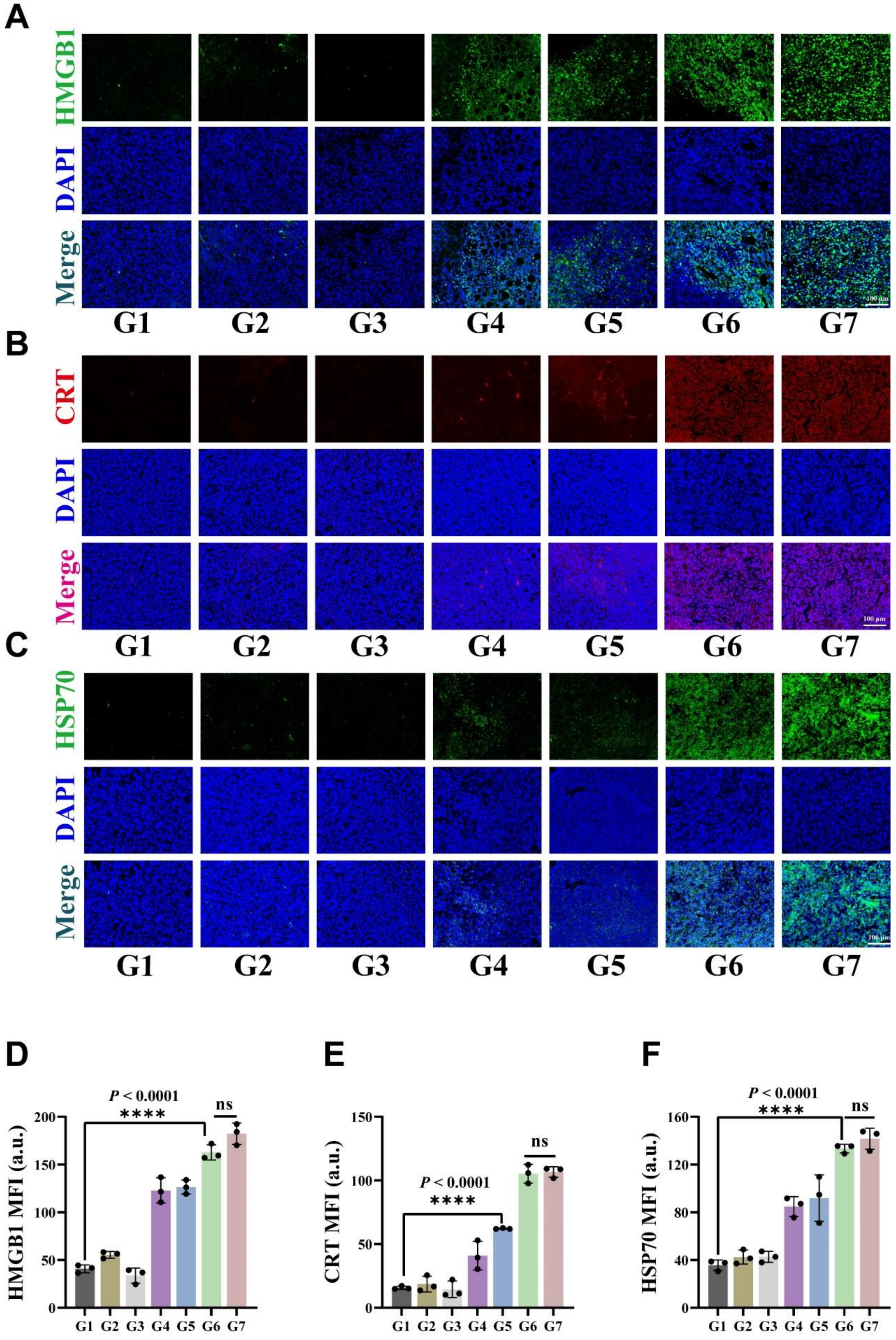
A
HMGB1
DAPI
Merge
G1
G2
G3
G4
G5
G6
G7
B
CRT
DAPI
Merge
G1
G2
G3
G4
G5
G6
G7
C
HSP70
DAPI
Merge
G1
G2
G3
G4
G5
G6
G7
D
HMGB1 MFI (a.u.)
P < 0.0001
****
ns
E
CRT MFI (a.u.)
P < 0.0001
****
ns
F
HSP70 MFI (a.u.)
P < 0.0001
****
ns

Figure S23. A) Different channels of immunofluorescence images of HMGB1 and B) CRT and C) HSP70 of tumor tissue after different treatment. D) Corresponding fluorescence intensity of HMGB1 and E) CRT and F) HSP70 proteins (*n* = 3). Control (G1), PSB@$Nb_{1.33}C$/mRNA (G2), laser (G3), $Nb_{1.33}C$/mRNA + laser (G4), PSB + laser (G5), PSB@$Nb_{1.33}C$ + laser (G6), PSB@$Nb_{1.33}C$/mRNA + laser (G7). *$P < 0.05$, **$P < 0.01$, ***$P < 0.001$, ****$P < 0.0001$.

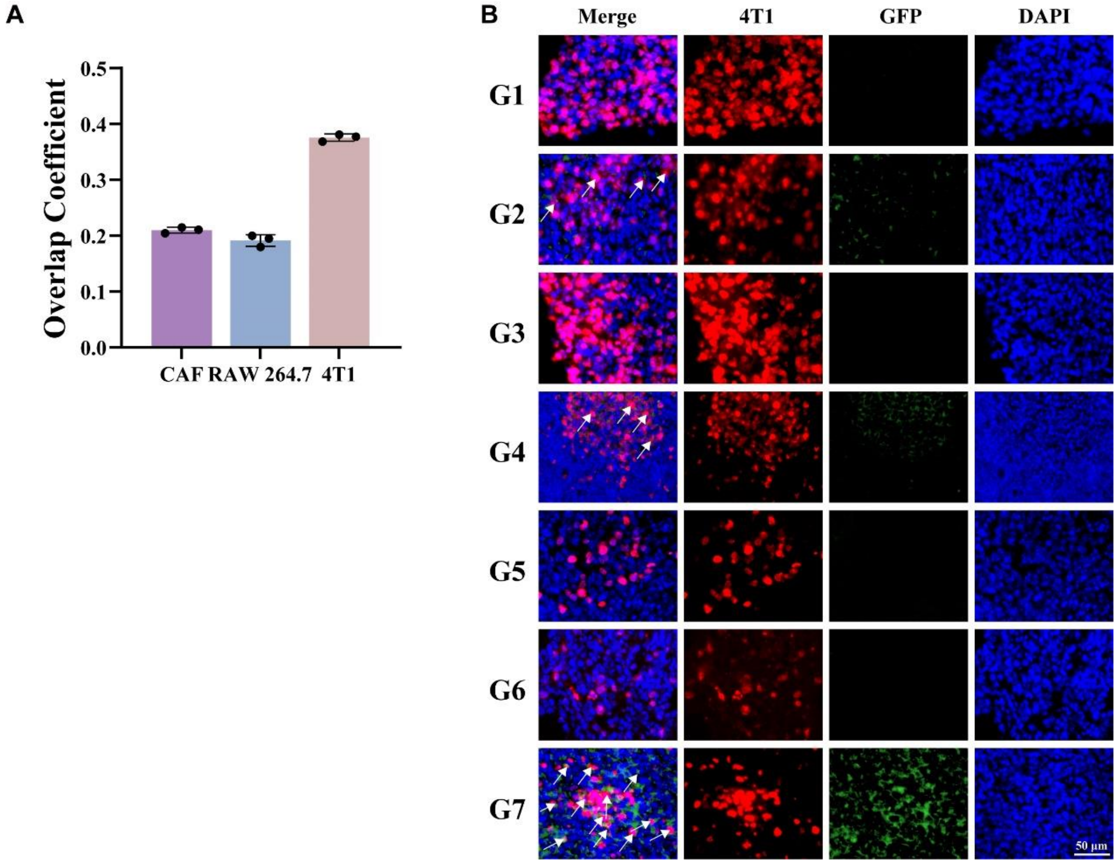

Figure S24. A) Fluorescence overlap coefficient between CAF (α-SMA positive), RAW 264.7 (F4/80 positive), 4T1 (Ki-67 positive area) and GFP positive area in tumor tissue (*n* = 3). B) Immunofluorescence images of GFP, 4T1 cells (Ki-67 positive) in tumor tissue after different treatments. Control (G1), PSB@$Nb_{1.33}C$/mRNA (G2), laser (G3), $Nb_{1.33}C$/mRNA + laser (G4), PSB + laser (G5), PSB@$Nb_{1.33}C$ + laser (G6), PSB@$Nb_{1.33}C$/mRNA + laser (G7).

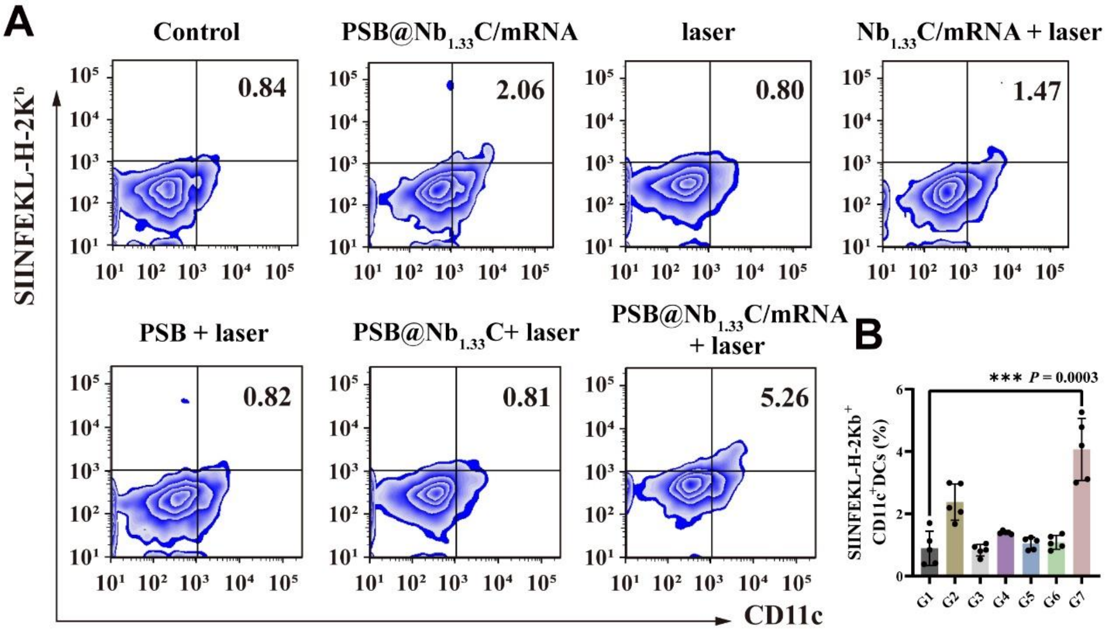

Figure S25. A) Typical FCM showing CD11c$^+$SIINFEKL$^+$ SIINFEKL-presenting DCs and B) quantitative analysis in the lymph nodes (n = 5). Control (G1), PSB@$Nb_{1.33}$C/mRNA (G2), laser (G3), $Nb_{1.33}$C/mRNA + laser (G4), PSB + laser (G5), PSB@$Nb_{1.33}$C + laser (G6), PSB@$Nb_{1.33}$C/mRNA + laser (G7). *$P < 0.05$, **$P < 0.01$, ***$P < 0.001$, ****$P < 0.0001$.

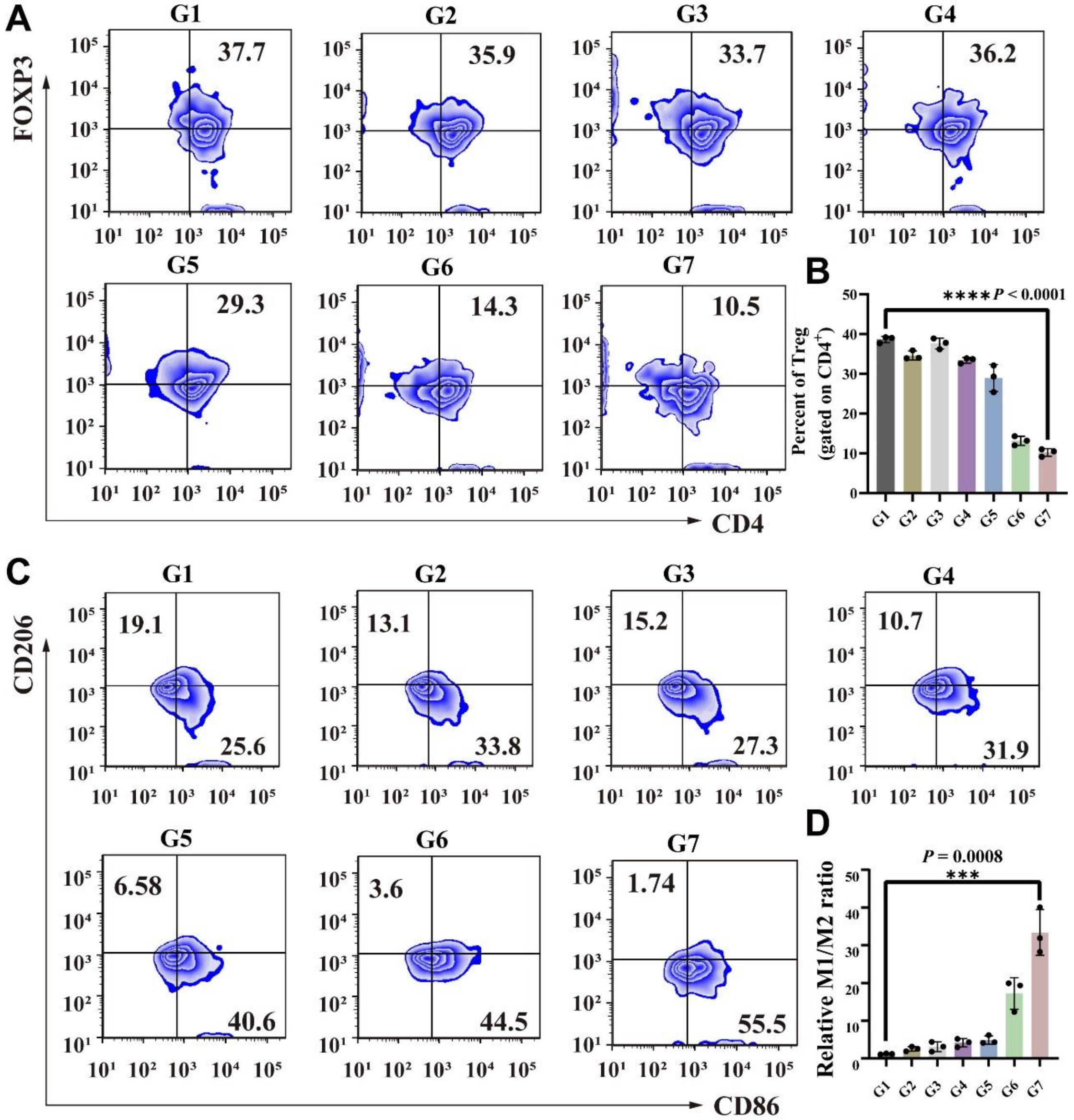

Figure S26. A) Representative flow cytometric and B) the quantitative analysis of Tregs in the primary 4T1 tumor tissue after the first different treatments (n = 3). C) Representative flow cytometric and D) the quantitative analysis of M2 macrophages in the primary 4T1 tumor tissue after the first different treatments (n = 3). Control (G1), PSB@$Nb_{1.33}C$/mRNA (G2), laser (G3), $Nb_{1.33}C$/mRNA + laser (G4), PSB + laser (G5), PSB@$Nb_{1.33}C$ + laser (G6), PSB@$Nb_{1.33}C$/mRNA + laser (G7). *$P < 0.05$, **$P < 0.01$, ***$P < 0.001$, ****$P < 0.0001$.

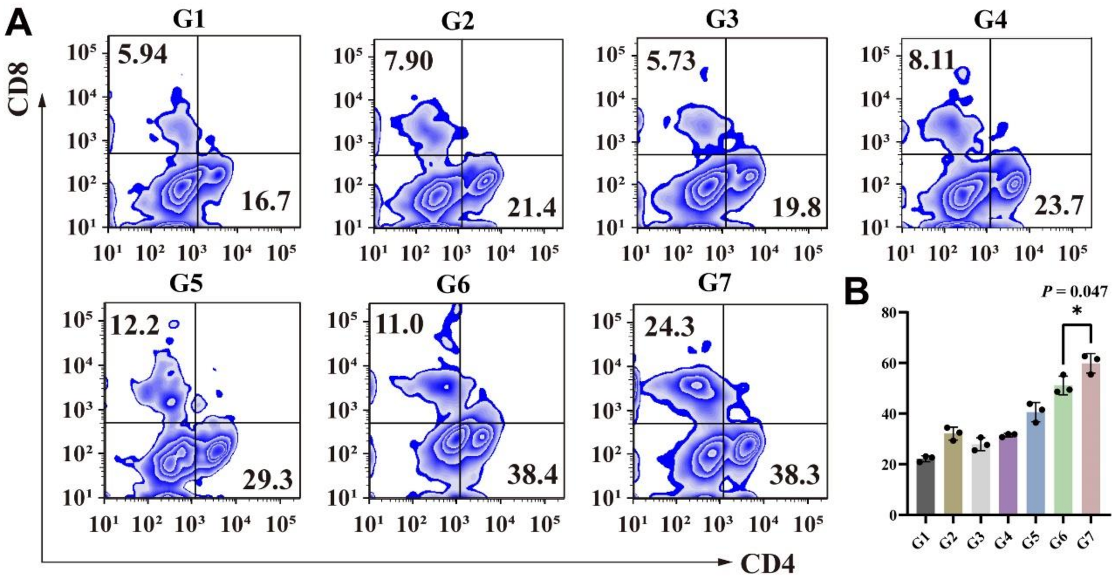

Figure S27. A) Representative flow cytometric and B) the quantitative analysis of T cells of $CD4^+$ and $CD8^+$ T cells in the LNs after the first different treatments (*n* = 3). Control (G1), PSB@$Nb_{1.33}C$/mRNA (G2), laser (G3), $Nb_{1.33}C$/mRNA + laser (G4), PSB + laser (G5), PSB@$Nb_{1.33}C$ + laser (G6), PSB@$Nb_{1.33}C$/mRNA + laser (G7). *$P < 0.05$, **$P < 0.01$, ***$P < 0.001$, ****$P < 0.0001$.

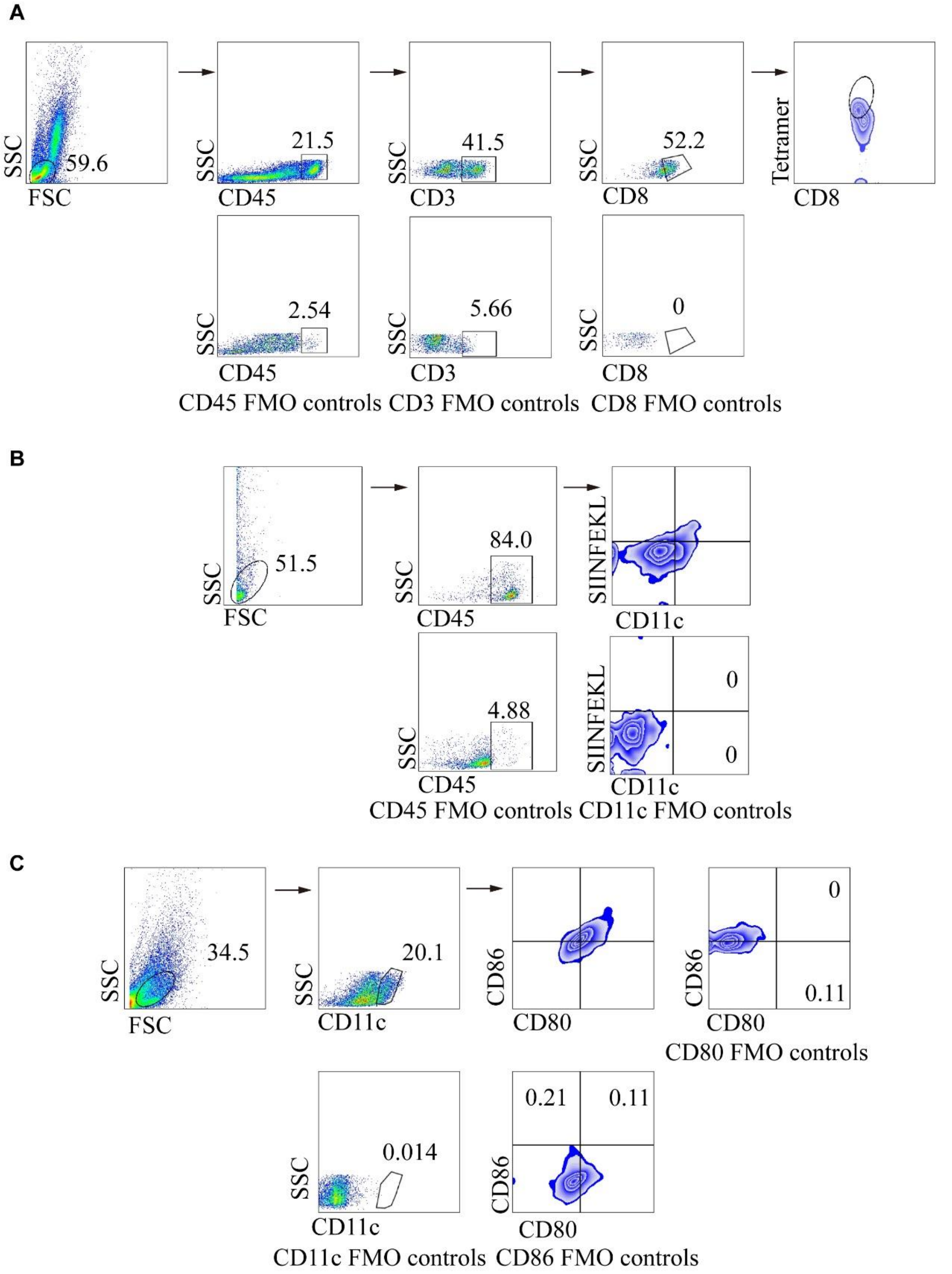

Figure S28. A) Flow cytometry gating strategy for the analysis of Tetramer^{+} CD8^{+} T cells. B) Flow cytometry gating strategy for the analysis of CD11c^{+} SIINFEKL^{+} presenting DCs. C) Flow cytometry gating strategy for the analysis of DCs in tumor.

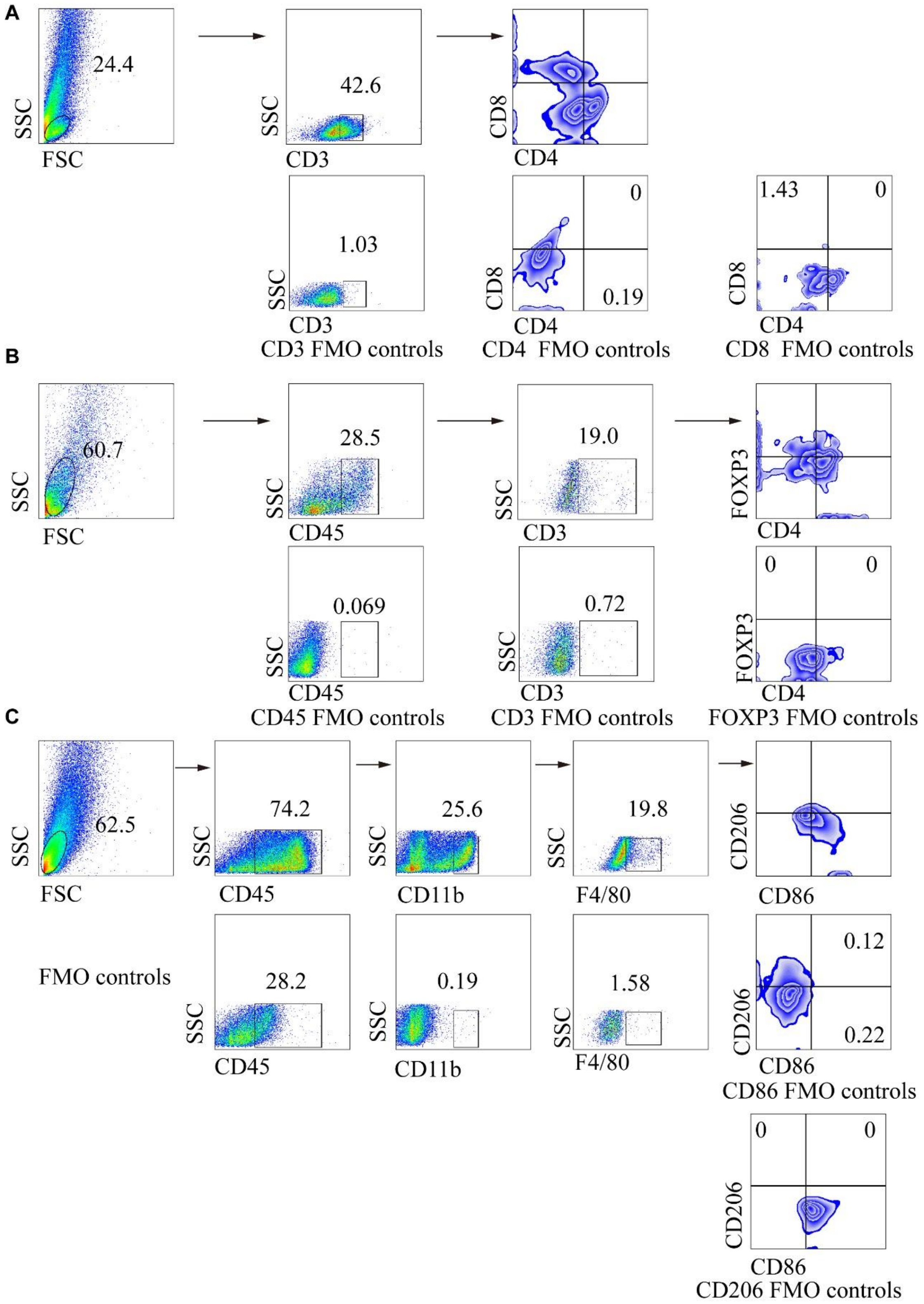

Figure S29. A) Flow cytometry gating strategy for the analysis of $CD4^+$ and $CD8^+$ T cells in primary tumor tissue. B) Flow cytometry gating strategy for the analysis of Tregs in tumor tissue. C) Flow cytometry gating strategy for the analysis of M2 macrophages in tumor.

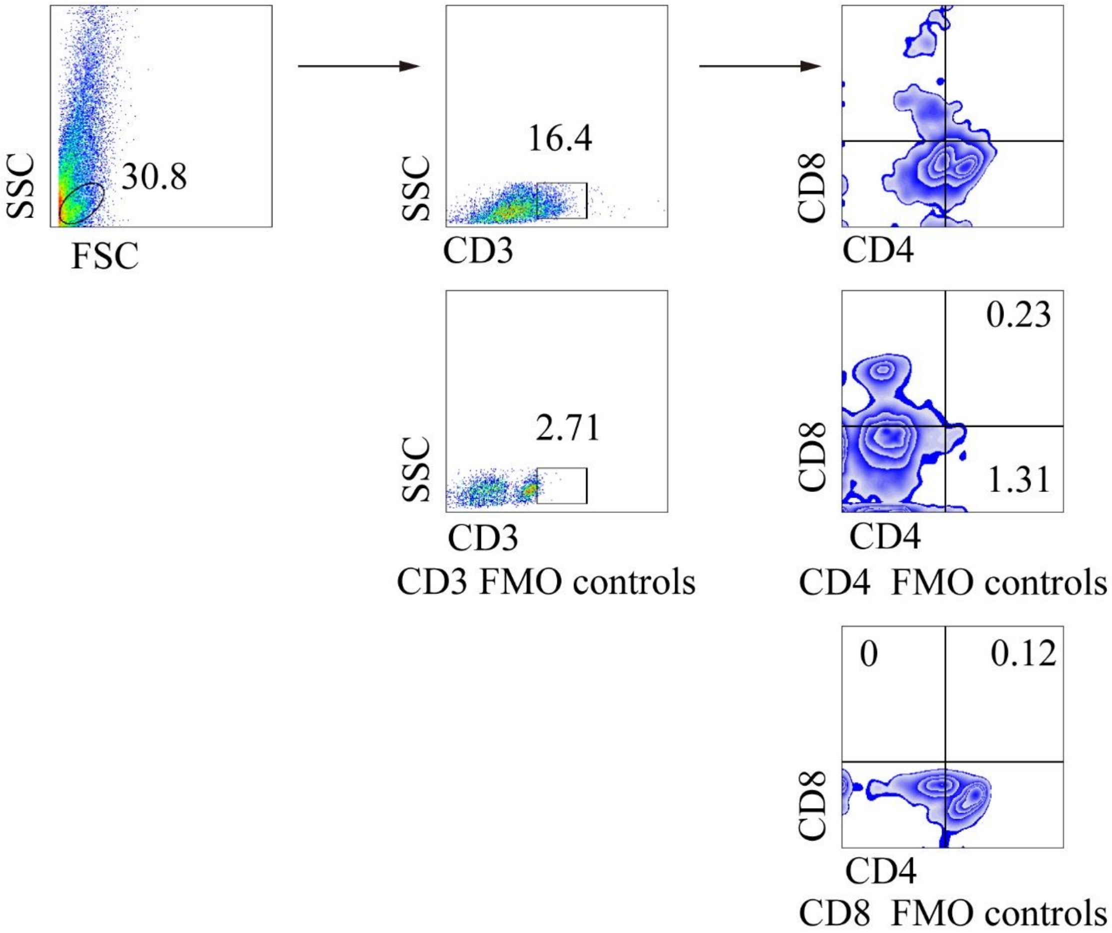

Figure S30. Flow cytometry gating strategy for the analysis of T cells of CD4$^+$ and CD8$^+$ T cells in the lymph nodes.

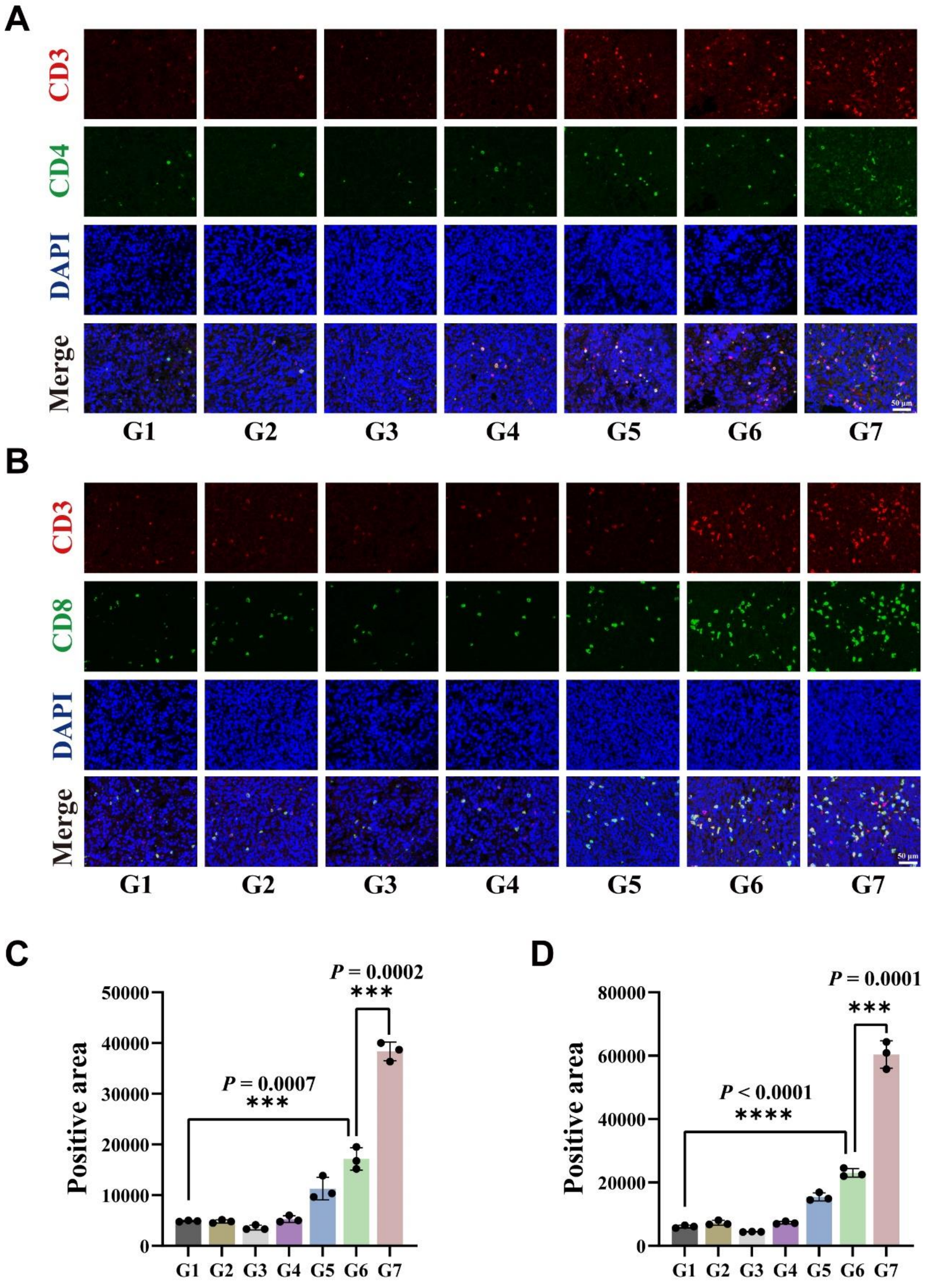

Figure S31. A) Different channels of immunofluorescence images of $CD3^+CD4^+$ and B) $CD3^+CD8^+$ proliferating CTLs in the primary tumor tissue sections after various treatments. C) Corresponding positive area quantification of $CD3^+CD4^+$ and D) $CD3^+CD8^+$ proliferating CTLs in the primary

tumor tissue sections after various treatments (n = 3). Control (G1), PSB@$Nb_{1.33}C$/mRNA (G2), laser (G3), $Nb_{1.33}C$/mRNA + laser (G4), PSB + laser (G5), PSB@$Nb_{1.33}C$ + laser (G6), PSB@$Nb_{1.33}C$/mRNA + laser (G7). *$P < 0.05$, **$P < 0.01$, ***$P < 0.001$, ****$P < 0.0001$.

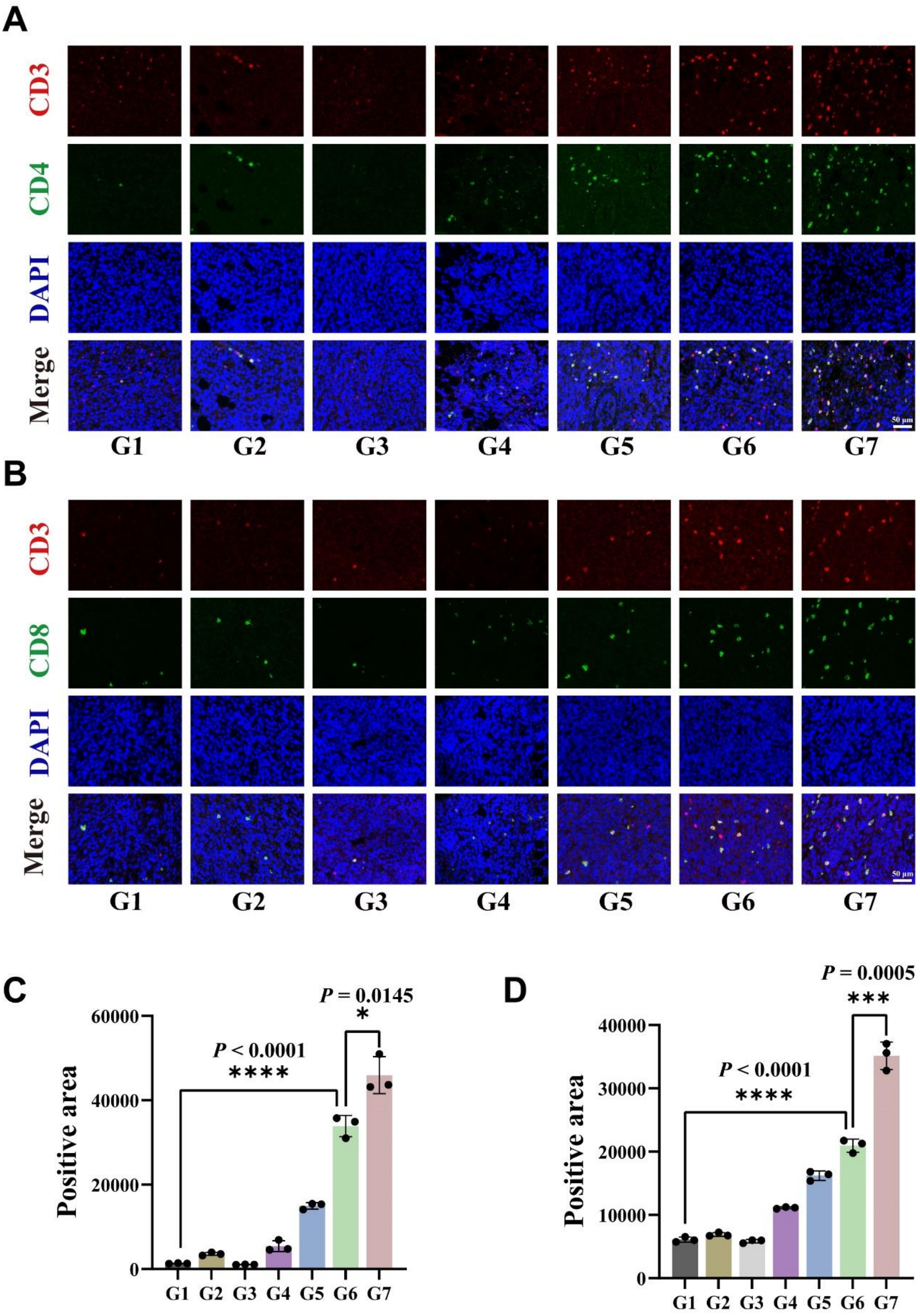

Figure S32. A) Different channels of immunofluorescence images of $CD3^{+}CD4^{+}$ and B) $CD3^{+}CD8^{+}$ proliferating CTLs for the distant tumor tissue sections after various treatments. C) Corresponding positive area quantification of $CD3^{+}CD4^{+}$ and D) $CD3^{+}CD8^{+}$ proliferating CTLs in the distant tumor

(n = 3). Control (G1), PSB@$Nb_{1.33}C$/mRNA (G2), laser (G3), $Nb_{1.33}C$/mRNA + laser (G4), PSB + laser (G5), PSB@$Nb_{1.33}C$ + laser (G6), PSB@$Nb_{1.33}C$/mRNA + laser (G7). *$P < 0.05$, **$P < 0.01$, ***$P < 0.001$, ****$P < 0.0001$.

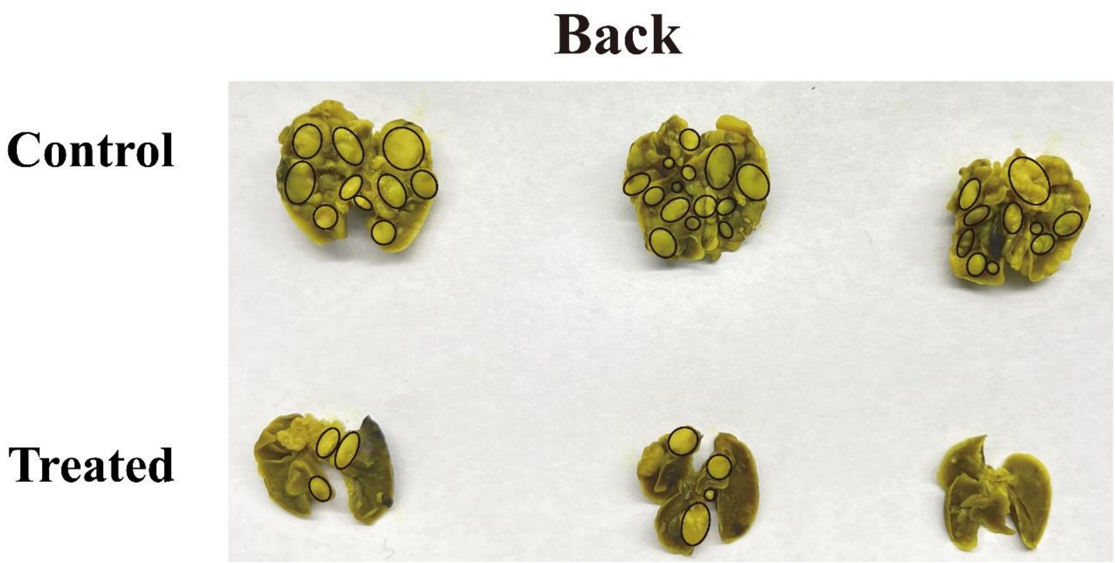

Figure S33. Representative photographs of lung metastatic nodules in the back for different groups (Control, Treated) (n = 3).
